# Role of the transmembrane domain in severe acute respiratory syndrome (SARS) coronavirus 2 spike for palmitoylation and membrane fusion

**DOI:** 10.1002/pro.70482

**Published:** 2026-01-23

**Authors:** Dina A. Abdulrahman, Michael Veit

**Affiliations:** ^1^ Institute of Virology, Department of Veterinary Medicine Freie Universität Berlin Berlin Germany; ^2^ Department of Virology Animal Health Research Institute Giza Egypt

**Keywords:** AlphaFold prediction, DHHC20, membrane fusion, mutational analysis, palmitoylation, post‐translational modification, SARS‐CoV‐2 spike, transmembrane domain

## Abstract

Palmitoylation is a reversible post‐translational modification that enhances protein hydrophobicity and regulates cellular functions such as trafficking and signaling. In humans, this modification is catalyzed by 23 DHHC enzymes, but the mechanisms by which they recognize their substrates remain unclear. The severe acute respiratory syndrome coronavirus 2 (SARS‐CoV‐2) spike protein undergoes palmitoylation primarily by DHHC20 with subsequent modification by DHHC9 at 10 cytoplasmic tail (CT) cysteines, a modification crucial for membrane fusion and viral entry. Using AlphaFold2 modeling and site‐directed mutagenesis, we identified three key components critical for efficient spike palmitoylation: (i) Lys1211 at the ectodomain–transmembrane domain (TMD) interface, likely facilitating electrostatic interactions with DHHC20's acidic residues; (ii) a stable trimeric TMD helix, where mutations at the trimer interface impair palmitoylation, in contrast to changes in outward‐facing residues; and (iii) a conserved hydrophilic motif in the CT, located between acylated cysteine clusters, likely promoting optimal substrate positioning near DHHC20's catalytic site. Co‐immunoprecipitation assays revealed that mutations in these residues disrupt spike‐DHHC20 interactions, while leaving spike–DHHC9 binding unchanged, suggesting that they affect enzyme‐substrate complex formation. Fusion assays revealed nuanced effects; while palmitoylation generally correlated positively with membrane fusion, certain exceptions highlighted the complex relationship between these processes. Mutations in the CT markedly reduce total spike palmitoylation but only modestly affect cell–cell fusion. Some substitutions in the TMD impair fusion with little change in overall acylation. Our findings elucidate the structural and biophysical determinants of spike palmitoylation and its distinct roles in membrane fusion, offering insights into SARS‐CoV‐2 pathogenesis and potential antiviral targets.

## 1 INTRODUCTION

Protein S‐acylation, commonly known as palmitoylation, is a reversible post‐translational modification that covalently attaches fatty acids, predominantly palmitate, to specific cysteine residues in proteins. This modification enhances protein hydrophobicity, promoting membrane association and modulating critical functions such as trafficking, localization, and signaling (Blaskovic et al., 2014). Aberrant palmitoylation is implicated in various diseases, including cancer, neurological disorders, and immune deficiencies (Mesquita et al., 2024). Additionally, pathogens such as viruses, bacteria, and parasites exploit the host's palmitoylation machinery to sustain their life cycles (Sobocińska et al., 2018).

Palmitoylation is catalyzed by DHHC enzymes, polytopic membrane proteins characterized by the conserved Asp‐His‐His‐Cys (DHHC) motif within a cysteine‐rich domain (CRD) (Linder & Jennings, 2013). Twenty‐three DHHC enzymes exist in humans, which localize mainly to the endoplasmic reticulum (ER) and Golgi, some also to the plasma membrane and endosomes (Zaballa & Gisou van der Goot, 2018). DHHC enzymes operate via a two‐step mechanism: auto‐acylation transfers a fatty acid from acyl‐CoA to the enzyme's DHHC motif, followed by its transfer to the substrate protein. Proteins are palmitoylated by specific DHHC enzymes, indicating distinct yet partially overlapping substrate specificities (Malgapo & Linder, 2021). While no universal consensus motif governs substrate recognition, individual DHHC enzymes likely bind substrates through specific amino acid patterns (Greaves & Chamberlain, 2011). Identifying amino acids that influence acylation could reveal recognition patterns, facilitating DHHC‐targeted interventions (Gadalla & Veit, 2020; Rana et al., 2018).

The discovery of protein palmitoylation originated with viral glycoproteins, which have been instrumental in elucidating its fundamental mechanisms (Blanc et al., 2013; Schmidt & Schlesinger, 1979). Palmitoylation of membrane‐proximal cysteines is essential for the function of certain viral glycoproteins and thus for virus replication (Chen et al., 2005; Thorp et al., 2006; Zhang et al., 2021). However, in some functionally similar glycoproteins, removal of palmitoylation sites has little effect on replication (Ito et al., 2001; Wang et al., 2016; Whitt & Rose, 1991). Viruses from diverse families employ distinct DHHC enzymes for glycoprotein palmitoylation, likely reflecting adaptations to a specific host environment (Abdulrahman et al., 2021).

The severe acute respiratory syndrome coronavirus 2 (SARS‐CoV‐2) spike protein, a homotrimeric type I transmembrane protein, comprises an ectodomain, a transmembrane region, and a short cytoplasmic tail (CT) (Li et al., 2022; Wrapp et al., 2020). The spike initiates viral infection by binding to the Angiotensin‐Converting Enzyme 2 (ACE2) receptor on host cells and mediating fusion between the viral envelope and host cell membrane (Hoffmann et al., 2020; Rajah et al., 2022). Palmitoylation occurs on 10 highly conserved cysteine residues within the CT. Several DHHC enzymes palmitoylate the spike, with DHHC9 and DHHC20 playing a predominant role. In the ER, DHHC20 acylates the two membrane‐adjacent cysteines, which bear ~80% of the fatty acids. In the Golgi, DHHC9 and DHHC20 extend acylation to downstream cysteines (Mesquita et al., 2021). This modification enhances spike stability, membrane fusion activity, and viral infectivity (Shulla et al. 2009; McBride et al. 2010; Mesquita et al., 2021; Puthenveetil et al., 2021; Ramadan et al., 2022; Wu et al., 2021). The molecular mechanism by which DHHC20 recognizes the spike and initiates catalysis remains unclear, but the spike's transmembrane domain (TMD) likely plays a role. The TMD, an unusually long amino acid stretch, features an aromatic‐rich region followed by a hydrophobic segment (Aliper & Efremov, 2023). Mutations in the tryptophan‐rich domain or hydrophobic region in SARS‐CoV‐1 and SARS‐CoV‐2 viruses impair virus entry, though it is uncertain whether these defects stem from reduced acylation or direct effects on membrane fusion (Corver et al., 2009; Dadonaite et al., 2023; Lu et al., 2008; Ortiz‐Mateu et al., 2024).

To investigate the TMD's role in spike acylation, we employed bioinformatics‐based models to identify amino acids potentially involved in TMD stability and DHHC20 interaction. Mutational analysis revealed six critical residues: an ectodomain lysine, three residues stabilizing the trimeric helix, and two hydrophilic residues in the CT. We also examined the relationship between reduced acylation and membrane fusion. While residues influencing helix stability and the ectodomain lysine consistently impacted both processes, hydrophobic residues exposed on the trimeric helix's exterior had negligible effects on either. Notably, mutations in the CT significantly decreased acylation but had minimal impact on fusion. These findings elucidate the TMD's role in spike acylation and membrane fusion, advancing our understanding of coronavirus infectivity and highlighting potential targets for antiviral therapies aimed at disrupting spike‐mediated entry.

## 2 RESULTS

### 2.1 AlphaFold2 model of the C‐terminus of SARS‐CoV‐2 spike protein

**FIGURE 1:**
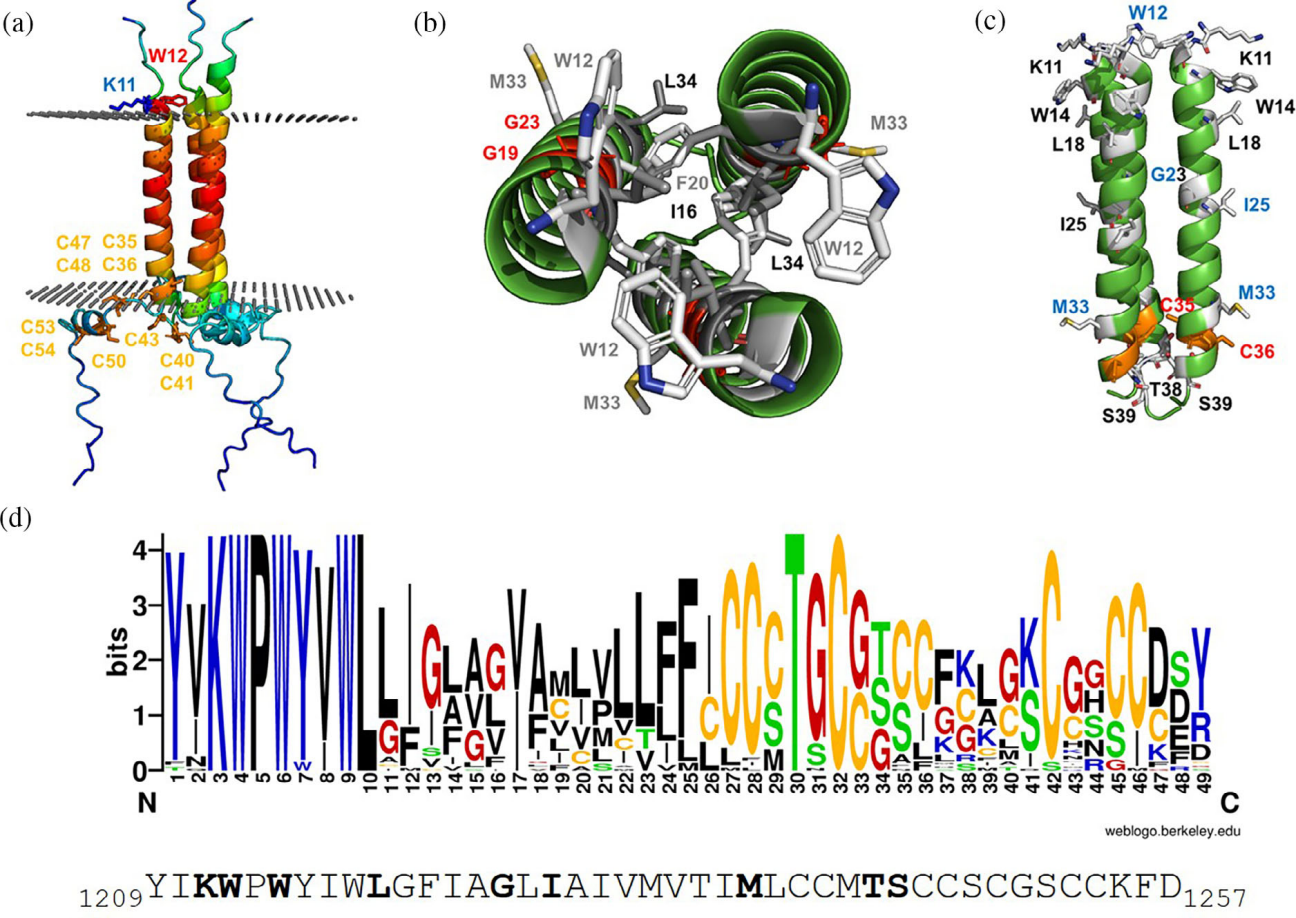
Structural analysis and conservation of the severe acute respiratory syndrome coronavirus 2 (SARS‐CoV‐2) spike protein C‐terminus. (a) Cartoon of the alphafold2 predicted structure embedded in a virtual membrane. The confidence score is color‐coded from red (high confidence) to blue (low confidence). Amino acids beyond position 1200 are designated by their single‐letter code followed by the last two digits of their position. The side chains of K11 and W12 are outside of the bilayer. The 10 acylated cysteines are shown as orange sticks. C35 and C 36 are the last residues within the lipid bilayer. (b) View from the N‐terminal part along the trimer helix bundle interior. Amino acids at the helix–helix interface, except for M33 which projects outward of the helical bundle, are shown as sticks and labeled. (c) Cartoon model of the TMR. The amino acids mutated in this study are shown as white sticks and the acylated cysteines at the end of the TMR as orange sticks. (d) Web logo showing the conservation of residues in the TMR of coronaviruses from all genera and various hosts. The sequence of the SARS‐CoV‐2 spike is shown below; amino acids exchanged in this study are in bold.

Using the CoLab AlphaFold2 multimer, we modeled the TMD and CT of the SARS‐CoV‐2 spike protein, positioning it in a virtual bilayer. Model confidence was assessed using the per‐residue predicted local distance difference test (pLDDT) scores and the predicted aligned error (PAE) matrix (Figure S1). AlphaFold2's pLDDT indicates high confidence (red, scores ~80) for the TMD's middle section and low confidence (blue, <50) for the CT, suggesting intrinsic disorder. K1211 and W1212 flank the N‐terminal part of the TMD, while C1235/36 are located at the transmembrane helix's end. All 10 acylated cysteine residues lie near the membrane, enabling lipid insertion (Figure 1a). Viewing the trimer helix bundle from the N‐terminus reveals that W1212, I1216, G1219, F1220, G1223, and L1234 are located at the helix–helix interface (Figure 1b). Molecular dynamics (MD) simulations also placed these residues at the helix–helix interface, except for M1233. While M1233's side chain projects outward from the helical bundle in the AlphaFold model (Figure 1b,c), it formed inter‐helical contacts during MD simulations (Aliper et al., 2022).

Analysis of TMD sequence conservation across 24 coronaviruses from various mammalian and avian hosts showed complete conservation of K1211, W1212, P1213, W1214, W1217, and L1218 in the N‐terminal region, along with T1238 and C1240 at the start of the CT. While the positions of other acylated cysteines vary, all coronaviruses have at least seven cysteines in this region. The middle TMD section is variable, with a conserved GXXXG motif in many coronaviruses (Figure 1d).

### 2.2 Design, expression, and trafficking of spike TMD and CT mutants

We designed two groups of mutants to probe the TMD's structure and function. The first group targets helix–helix interface residues, which are unlikely to bind DHHC enzymes directly but are critical for TMD stability. Three mutants were created: W1212A replaces tryptophan with alanine, weakening N‐terminal helix contacts; M1233A disrupts C‐terminal interactions, as suggested by MD simulations (Aliper et al., 2022); and G1223W introduces a bulky tryptophan, likely disrupting helical packing due to steric hindrance. Additionally, I1225A was included, as its outward‐projecting isoleucine is expected to minimally affect helix stability (Figure 1c).

The second group tests whether DHHC enzymes recognize a conserved motif on the helix's exterior, assuming shared acylation mechanisms across coronaviruses. We targeted K1211, W1214, and L1218, located on the same helix face, and W1212, which are all highly conserved. These residues were mutated either individually (K1211A) or in combination, including a construct with all four residues substituted, to assess both individual and collective effects on spike palmitoylation.

Finally, we mutated hydrophilic residues T1238 (100% conserved) and S1239 (sometimes glycine in coronaviruses) (Figure 1d), proposed to interact with DHHC20's hydrophilic patch (Panina et al., 2022). These were replaced with alanine individually, together, or in combination with the G1223W mutation. In total, 14 mutants were generated (Table 1).

**TABLE 1:** Summary of acylation assay, co‐immunoprecipitation (CO‐IP) with DHHC20, and syncytium formation assay results. Amino acids beyond position 1200 are designated by their single‐letter code followed by the last two digits of their position. Acylation levels, protein co‐precipitated with DHHC20, and fusion activity are normalized to wild‐type values. Reduction of acylation: 20–30: ++++, 30–40: +++, 40–50: ++, 50–60: +, 61–81: +/−. The second acylation values for the double and quadruple K11A mutants (italicized) were obtained in parallel with the single K11A mutant (Figure 4), while the second values (underlined) were determined in the presence of palmostatin B (Figure 6). Fusion activity: 19–35: ++++, 36–50: +++, 51–65: ++, 65–80: +, >80: +/−. n.a., not analyzed; ns, not significant.

Mutant	Acylation level	Co‐IP with DHHC 20	Co‐IP with DHHC 9	Fusion activity	Reduction of acylation	Reduction of fusion
W12A	61%	60%	n.a.	25%	+	++++
G23W	45%, 31%	47%	106% (ns)	40%	++, +++	+++
I25A	76% (ns)	80% (ns)	n.a.	81%	+/−	+/−
M33A	52%	35%	n.a.	70%	+	+
WM12/33AA	36%, 35%	24%	98% (ns)	20%	+++	++++
WW12/14AA	55%	n.a.	n.a.	30%	+	++++
WL14/18AA	81% (ns)	102% (ns)	133% (ns)	43%	+/−	+++
K11A	25%, 40%	n.a.	n.a.	n.a.	++++, +++	n.a.
KL11/18AA	35%,*34%*	76%	n.a.	43%	+++, +++	+++
KW11/14AA	25%, *34%*	n.a.	83% (ns)	48%	++++, +++	+++
KWWL4A	37%, *26%*	n.a.	n.a.	19%	+++, ++++	++++
T38A	49%	n.a.	n.a.	73%	++	+
S39A	52%	n.a.	n.a.	128%	+	‐
TS38/39AA	33%, 41%	35%	113% (ns)	56%	+++, ++	++
GW/TS2A	28%	n.a.	n.a.	36%	++++	+++

**FIGURE 2:**
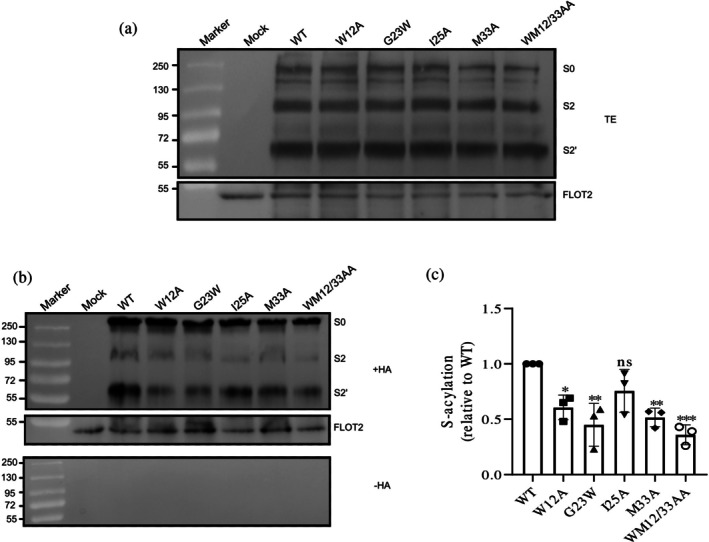
Impact of transmembrane domain mutations on spike palmitoylation (Set 1: residues potentially impacting helix stability). (a) Western blot analysis of total protein extracts (TE): 10% of each sample used to evaluate the expression levels of spike mutants relative to wild‐type (WT). Membranes were probed with anti‐spike‐S2 and anti‐flotillin‐2 antibodies. Full‐length spike (S0) and its cleavage products (S2 and S2′) are indicated. Flotillin‐2 (FLOT2), an endogenous cellular protein, serves as a loading control. The mock lane represents non‐transfected cells. Molecular weight markers are displayed on the left. (b) S‐acylation analysis: Samples were treated with either hydroxylamine (+HA) to cleave cysteine‐bound fatty acids or Tris–HCl (−HA) to assess the specificity of the acylation test. The banding pattern is as described in (a), with FLOT2 as a loading control. (c) Quantification of the acylation levels: Normalizing the band densities of the mutants to their respective input bands and comparing them to WT (set to 1). Data are shown as the mean ± SD from three independent experiments. Statistical analysis was conducted using one‐way analysis of variance followed by Dunnett's multiple comparison test. ns, not significant *p < 0.05, **p < 0.01, ***p < 0.001.

**FIGURE 3:**
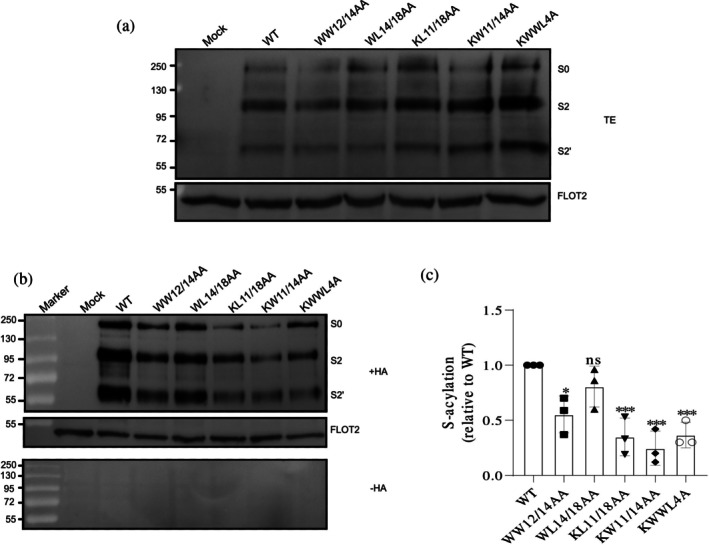
Impact of transmembrane domain mutations on spike palmitoylation (Set 2: conserved surface‐exposed residues). (a) Western blot analysis of total protein extracts (TE): 10% of each sample used to evaluate the expression levels of spike mutants relative to WT. Membranes were probed with anti‐spike‐S2 and anti‐flotillin‐2 antibodies. Full‐length spike (S0) and its cleavage products (S2 and S2′) are indicated. Flotillin‐2 (FLOT2), an endogenous cellular protein, serves as a loading control. The mock lane represents non‐transfected cells. Molecular weight markers are displayed on the left. (b) S‐acylation analysis: Samples were treated with either hydroxylamine (+HA) to cleave cysteine‐bound fatty acids or Tris–HCl (−HA) to assess the specificity of the acylation test. The banding pattern is as described in (a), with FLOT2 as a loading control. (c) Quantification of the acylation levels: Normalizing the band densities of the mutants to their respective input bands and comparing them to wild‐type (WT) (set to 1). Data are shown as the mean ± SD from three independent experiments. Statistical analysis was conducted using one‐way analysis of variance followed by Dunnett's multiple comparison test. ns, not significant, *p < 0.05, ***p < 0.001.

**FIGURE 4:**
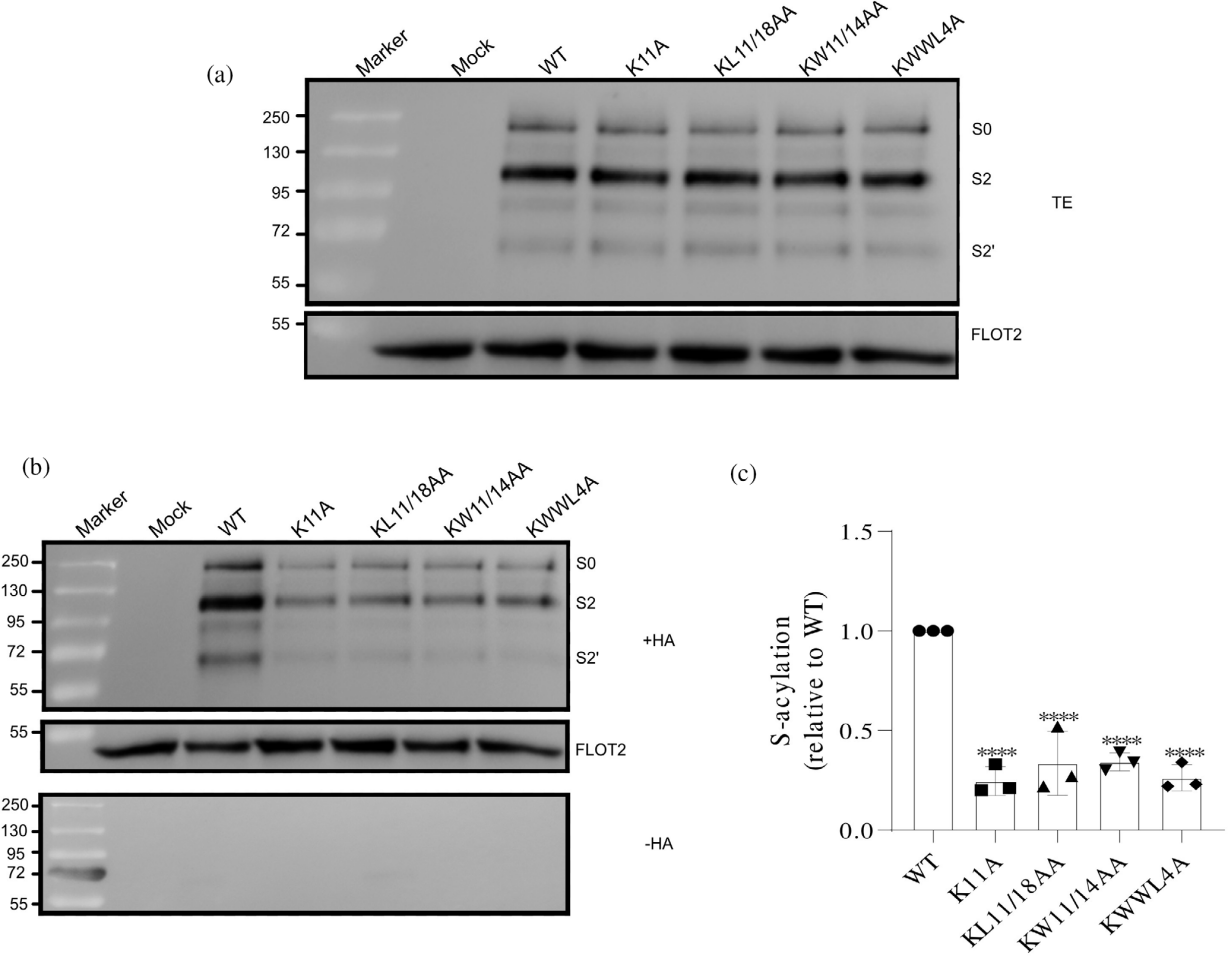
Impact of K1211A single mutation on spike palmitoylation. (a) Western blot analysis of total protein extracts (TE): 10% of each sample used to evaluate the expression levels of spike mutants relative to wild‐type (WT). Membranes were probed with anti‐spike‐S2 and anti‐flotillin‐2 antibodies. Full‐length spike (S0) and its cleavage products (S2 and S2′) are indicated. Flotillin‐2 (FLOT2), an endogenous cellular protein, serves as a loading control. The mock lane represents non‐transfected cells. Molecular weight markers are displayed on the left. (b) S‐acylation analysis: Samples were treated with either hydroxylamine (+HA) to cleave cysteine‐bound fatty acids or Tris–HCl (−HA) to assess the specificity of the acylation test. The banding pattern is as described in (a), with FLOT2 as a loading control. (c) Quantification of the acylation levels: Normalizing the band densities of the mutants to their respective input bands and comparing them to WT (set to 1). Data are shown as the mean ± SD from three independent experiments. Statistical analysis was conducted using one‐way analysis of variance followed by Dunnett's multiple comparison test. ****p < 0.0001.

**FIGURE 5:**
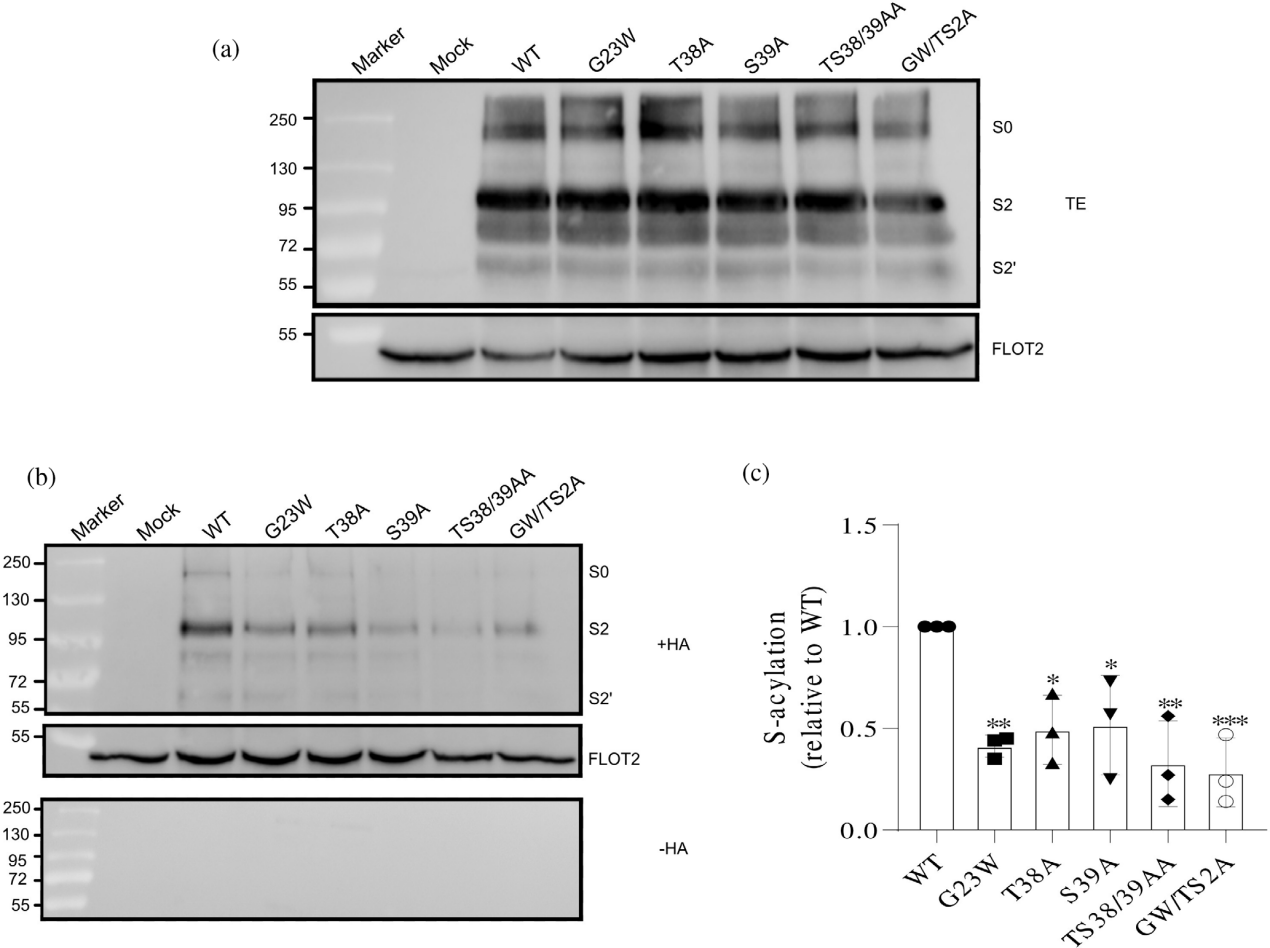
Impact of cytoplasmic tail mutations on spike palmitoylation (Set 3: hydrophilic, potentially DHHC20‐interacting residues). (a) Western blot analysis of total protein extracts (TE): 10% of each sample used to evaluate the expression levels of spike mutants relative to wild‐type (WT). Membranes were probed with anti‐spike‐S2 and anti‐flotillin‐2 antibodies. Full‐length spike (S0) and its cleavage products (S2 and S2′) are indicated. Flotillin‐2 (FLOT2), an endogenous cellular protein, serves as a loading control. The mock lane represents non‐transfected cells. Molecular weight markers are displayed on the left. (b) S‐acylation analysis: Samples were treated with either hydroxylamine (+HA) to cleave cysteine‐bound fatty acids or Tris–HCl (−HA) to assess the specificity of the acylation test. The banding pattern is as described in (a), with FLOT2 as a loading control. (c) Quantification of the acylation levels: Normalizing the band densities of the mutants to their respective input bands and comparing them to WT (set to 1). Data are shown as the mean ± SD from three independent experiments. Statistical analysis was conducted using one‐way analysis of variance followed by Dunnett's multiple comparison test. *p < 0.05, **p < 0.01, ***p < 0.001. Note the variability in S0, S2, and S2′ band patterns across Figures 2, 3, 4, 5, reflecting experiment‐specific differences in spike protein cleavage, which do not affect the quantitative evaluation of the acylation data.

Western blot analysis showed that the expression levels of spike proteins in all mutants were comparable to those of the WT spike (Figures 2a, 3a, 4a, and 5a). To assess surface transport, flow cytometry was used to calculate the ratio of surface‐expressed to intracellular spike protein, revealing no significant defects in trafficking for the mutants compared to the WT (Figure S2).

### 2.3 Effect of TMD and CT mutations on spike protein palmitoylation

To assess palmitoylation in transfected HEK 293T cells, we performed an acyl‐RAC (resin‐assisted capture) assay, which exploits thiol‐reactive resins to capture SH groups in proteins. Transfected cells were lysed and 10% of the total extract (TE) was removed to determine the expression levels (Figures 2a, 3a, 4a, and 5a). Disulfide bonds in proteins present in the remaining part were reduced and newly exposed ‐SH groups were blocked. The sample was then equally split: one aliquot was treated with hydroxylamine (+HA) to cleave thioester bonds, and the other aliquot was treated as control with Tris–HCl buffer (–HA). After the pull‐down of proteins with the thiol‐reactive resin, samples were subjected to Western blotting using antibodies against the S2 subunit of the spike protein. We quantified protein band intensities from three independent experiments for the mutants and normalized them to wild‐type (WT) levels (Figures 2b, 3b, 4b, and 5b).

The results of the mutations tested for their effect on helix stability showed that while all mutants retained detectable palmitoylation, four of the five exhibited significant reductions. The G1223W mutation had the strongest impact, decreasing palmitoylation by 55%. W1212A and M1233A mutations reduced acylation by 39% and 48%, respectively, and their combination led to a 64% reduction. In contrast, I1225A had only a minor, statistically insignificant effect (Figure 2c).

Quantification of the effect of conserved, surface‐exposed residues revealed that mutants KW1211/14AA and KL1211/18AA exhibited the most significant reductions, decreasing palmitoylation by 75% and 65%, respectively. This effect was primarily due to K1211 substitution, as mutations in W1214 and L1218 had little additional impact (Figure 3c). This conclusion was further supported by analysis of the single K1211A mutant, which alone recapitulated the extent of acylation loss observed in the corresponding double and quadruple mutants (Figure 4c). Similarly, residue W1214 is not crucial, since the WW1212/14AA mutant showed almost the same reduction in palmitoylation as the W1212A mutant alone, 45% versus 39%. Additionally, combining the L1218A mutation with the other three mutations in KWWL4A did not further decrease acylation (Figure 3c). Thus, we conclude that among the surface‐exposed residues, only K1211 has a significant effect on acylation, while W1214 and L1218 play a little role.

Finally, we assessed T1238 and S1239, hypothesized to interact with a hydrophilic stretch in DHHC20. T1238A and S1239A mutations each reduced palmitoylation by ~50% and up to 67% when combined, extending previous less precise data (Panina et al., 2022). To test the interplay between transmembrane and cytoplasmic determinants, we analyzed a triple mutant (GW/TS2A) that incorporates substitutions, G1223W, T1238A, and S1239A, in both domains. This variant showed the strongest reduction in acylation (~72%), modestly exceeding the double mutant, although residual fatty acid attachment remained detectable (Figure 5c).

**FIGURE 6:**
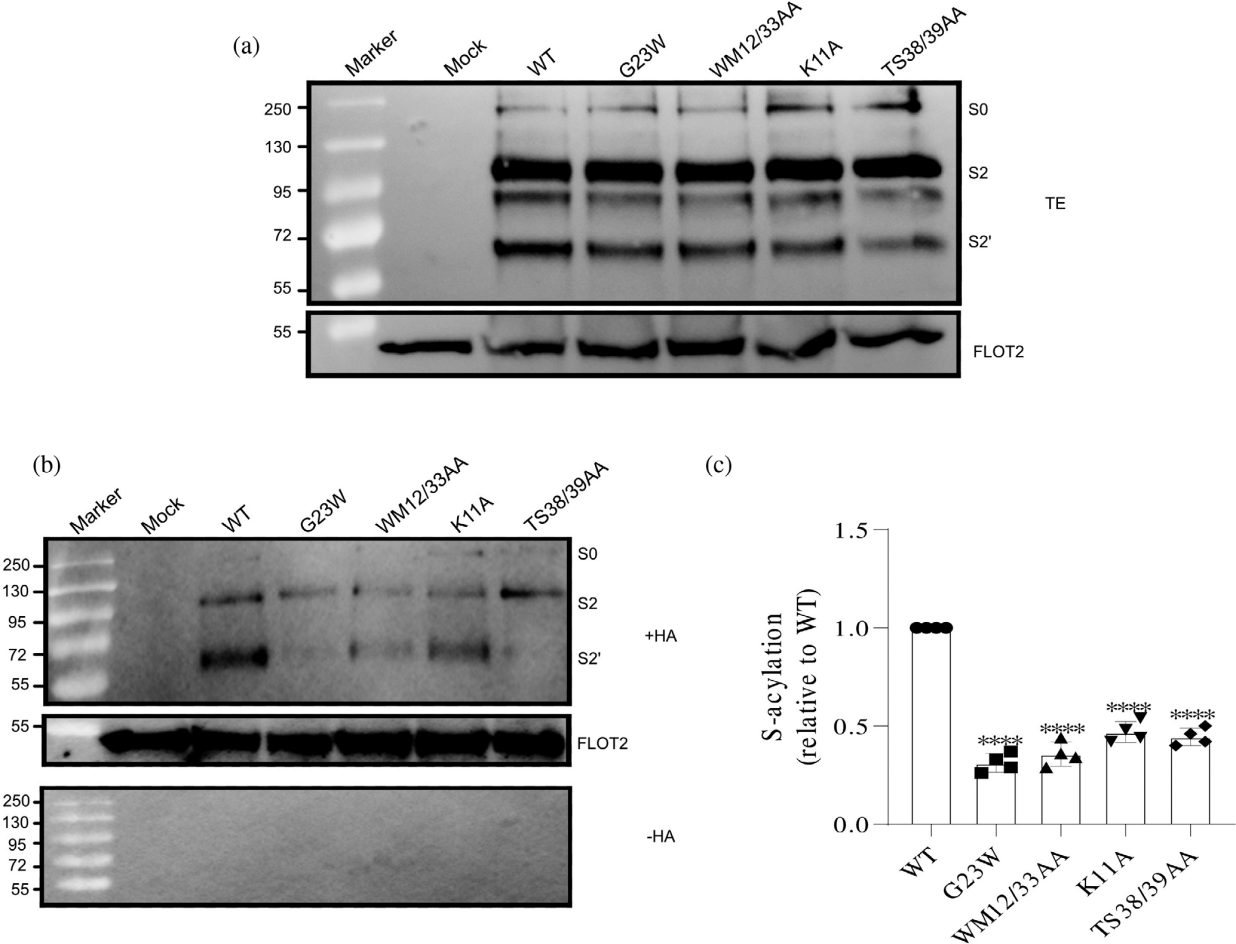
Impact of some mutations on spike palmitoylation under Palmostatin B treatment. (a) Western blot analysis of total protein extracts (TE): 10% of each sample was used to evaluate the expression levels of spike mutants relative to wild‐type (WT) after treatment with 37 μM of Palmostatin B. Membranes were probed with anti‐spike S2 and anti‐flotillin‐2 antibodies. Full‐length spike (S0) and its cleavage products (S2 and S2′) are indicated. Flotillin‐2 (FLOT2) served as a loading control. The mock lane represents non‐transfected cells. Molecular weight markers are shown on the left. (b) S‐acylation analysis: Samples were subjected to the acyl‐RAC assay following 37 μM Palmostatin B treatment. Hydroxylamine (+HA) was used to cleave cysteine‐bound fatty acids, whereas Tris–HCl (−HA) served as a negative control. The banding pattern corresponds to that in (a), with FLOT2 as a loading control. (c) Quantification of S‐acylation levels: Band intensities of mutant spike proteins were normalized to their respective input controls and expressed relative to WT (set to 1). Data represent mean ± SD from four independent experiments. Statistical analysis was performed using one‐way analysis of variance followed by Dunnett's multiple comparison test. ****p < 0.0001.

To determine whether the reduced acyl‐RAC signal observed in the spike mutants was due to increased deacylation rather than decreased acylation, we treated transfected cells with Palmostatin B, which inhibits the acyl‐protein thioesterase 1 (APT1). Increasing concentrations of Palmostatin B revealed increased palmitoylation of the spike, confirming that the spike is indeed depalmitoylated if expressed from a plasmid (Mesquita et al., 2021) (Figure S3). We then used the highest concentration of Palmostatin B that did not cause significant cell death to analyze four spike mutants (G123W, WM1212/33AA, K1211A, and TS1238/39AA), which exhibited a strong reduction in acylation in the absence of Palmostatin B. Even under continuous inhibition of depalmitoylation, these mutant spike proteins displayed markedly lower acylation levels compared to WT spike (Figure 6a–c), comparable to the reduction seen without Palmostatin B treatment. These results confirm that the observed decrease in acylation in spike mutants is primarily due to impaired acylation, not to accelerated turnover or enhanced deacylation.

### 2.4 Effect of TMD and CT mutations on spike interaction with hDHHC20 and mDHHC9

**FIGURE 7:**
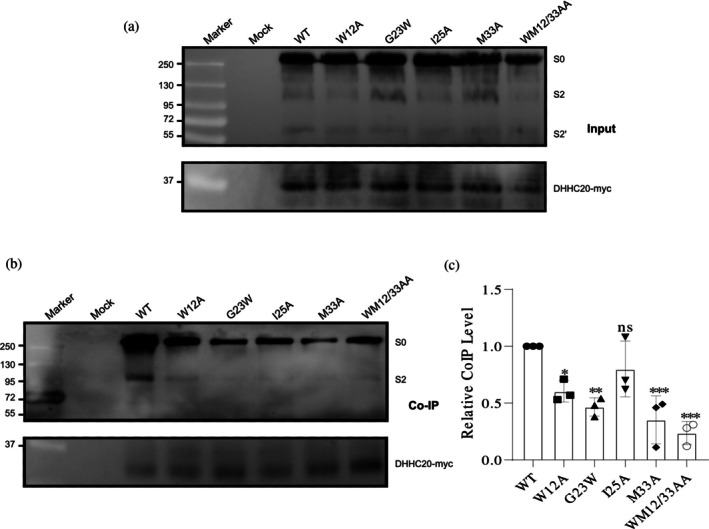
Co‐precipitation of hDHHC20 with wild‐type (WT) and mutant spikes (first set: residues potentially impacting helix stability) (a) Input: Expression of WT and the spike mutants, along with hDHHC20‐myc, was assessed by Western blotting using anti‐myc and anti‐S2 subunit antibodies. S0 represents the full‐length spike while S2 and S2′ are its cleavage product. (b) Co‐immunoprecipitation (Co‐IP): The remainder of the lysate was subjected to immunoprecipitation using anti‐myc‐tag antibodies, followed by Western blotting with anti‐myc and anti‐S2 subunit antibodies. (c) Quantification of mutant spike protein interactions with hDHHC20. Band intensities of the mutant spikes were normalized to their respective hDHHC20 bands and compared to the normalized values of WT. Data represent the mean ± SD from three independent experiments. Statistical significance was determined using one‐way analysis of variance followed by Dunnett's multiple comparison test. ns, not significant; *p < 0.05, **p < 0.01, ***p < 0.001. Note that little of the cleaved spike is present in the total extract compared to the acyl‐RAC assay, likely because cells were lysed with non‐denaturing NP‐40, which may not efficiently solubilize cleaved spike protein.

**FIGURE 8:**
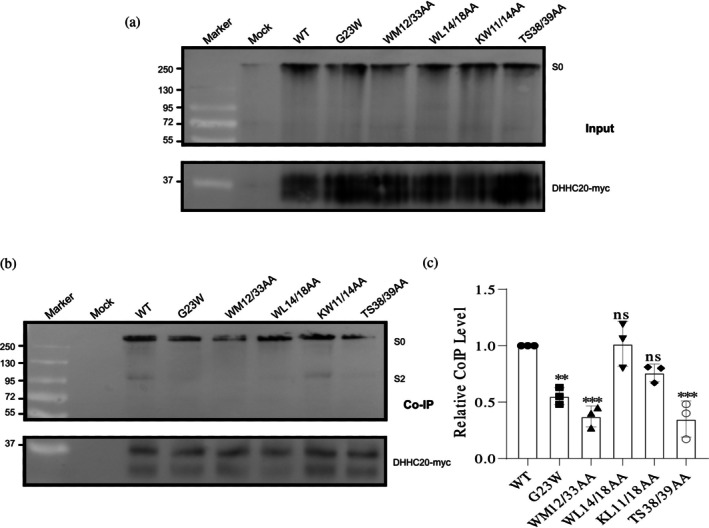
Co‐precipitation of hDHHC20 with wild‐type (WT) and mutant spikes (additional mutants with reduced acylation) (a) Input: Expression of WT and the spike mutants, along with hDHHC20‐myc, was assessed by Western blotting using anti‐myc and anti‐S2 subunit antibodies. S0 represents the full‐length spike. (b) Co‐IP: The remainder of the lysate was subjected to immunoprecipitation using anti‐myc‐tag antibodies, followed by Western blotting with anti‐myc and anti‐S2 subunit antibodies. (c) Quantification of mutant spike protein interactions with hDHHC20. Band intensities of the mutant spikes were normalized to their respective hDHHC20 bands and compared to the normalized values of WT. Data represent the mean ± SD from three independent experiments. Statistical significance was determined using one‐way analysis of variance followed by Dunnett's multiple comparison test. ns, not significant; **p < 0.01, ***p < 0.001.

The reduction in acylation could result from impaired enzyme–substrate complex formation or disruption of the subsequent fatty acid transfer. To investigate this, co‐immunoprecipitation (Co‐IP) assays were performed using hDHHC20‐myc and either WT or mutant spike proteins. The spike‐DHHC20 interaction was first confirmed with the WT spike to validate the assay before analyzing the mutants (Figure S4A,B). Western blot analysis confirmed expression of WT and mutated spike proteins alongside DHHC20 in the co‐transfected cells (Figures 7a and 8a), Spike‐hDHHC20 complexes were then immunoprecipitated using an anti‐myc‐tag antibody and detected by blotting with both anti‐S2 and anti‐myc antibodies. The ratios of co‐precipitated spike mutant proteins to their respective hDHHC20‐myc were calculated and normalized to the values obtained for the WT spike (Figures 7b and 8b).

The results showed varying degrees of spike‐hDHHC20 interaction. Three mutants—M1233A, WM1212/33AA, and TS1238/39AA—displayed the weakest interactions, with Co‐IP levels ranging from 24% (WM1212/33AA) to 35% (M1233A and TS1238/39AA) of WT. W1212A and G1223W mutants also exhibited reductions (60% and 47%, respectively), while I1225A and KL1211/18AA had only a slight impact (~20% reduction). The W1214A/L18AA mutant maintained WT‐like Co‐IP levels, indicating no effect on enzyme binding (Figures 7c and 8c).

The results from the acylation assays and Co‐IP experiments demonstrate a good correlation, suggesting that the observed reduction in acylation is primarily attributable to the diminished formation of the enzyme–substrate complex.

**FIGURE 9:**
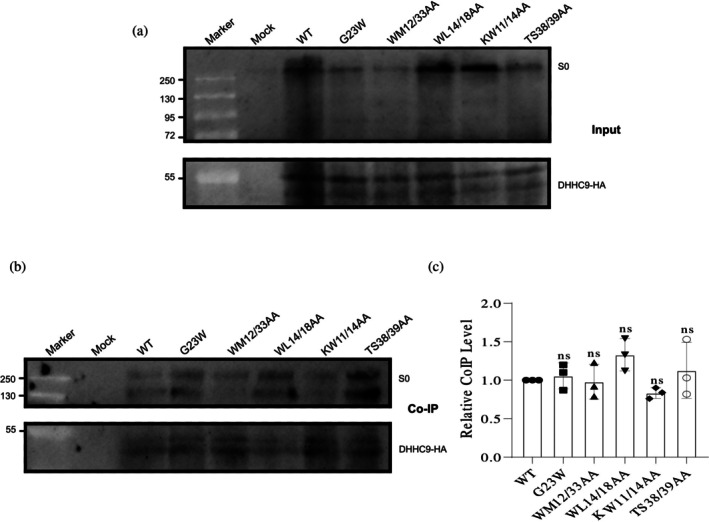
Co‐precipitation of mDHHC9 with wild‐type (WT) and mutant spikes: (a) Input: Expression of WT and the spike mutants, along with mDHHC9‐HA, was assessed by Western blotting using anti‐hydroxylamine (HA) and anti‐S2 subunit antibodies. S0 represents the full‐length spike. (b) Co‐immunoprecipitation (Co‐IP): The remainder of the lysate was subjected to immunoprecipitation using anti‐HA‐tag antibodies, followed by Western blotting with anti‐HA and anti‐S2 subunit antibodies. (c) Quantification of mutant spike protein interactions with mDHHC9. Band intensities of the mutant spikes were normalized to their respective mDHHC9 bands and compared to the normalized values of WT. Data represent the mean ± SD from three independent experiments. Statistical significance was determined using one‐way analysis of variance followed by Dunnett's multiple comparison test. ns, not significant.

To assess whether the mutations alter interaction with DHHC9, a spike‐acylating enzyme previously implicated in palmitoylation (Mesquita et al., 2021), we examined spike–DHHC9 association in a co‐transfection system. HA‐tagged mouse DHHC9 was used, sharing >95% sequence identity with the human ortholog and a fully conserved TMD; the few differences reside in the unstructured C‐terminal region and are unlikely to affect binding or activity. Co‐IP confirmed spike–DHHC9 interaction upon co‐expression (Figure S4C,D). Five spike mutants spanning the transmembrane region were tested. Three (G1223W, WM1212/33AA, and TS1238/39AA) showed markedly reduced interaction with DHHC20, one (WL1214/18AA) showed no reduction, and one (KW1211/14AA) had not been previously analyzed. In contrast, all mutants retained DHHC9 binding at levels comparable to WT (Figure 9a–c). These data indicate that TMD mutations do not impair spike recognition by DHHC9, suggesting that determinants outside the mutated region mediate this association.

### 2.5 Effect of reduced palmitoylation on spike membrane fusion

**FIGURE 10:**
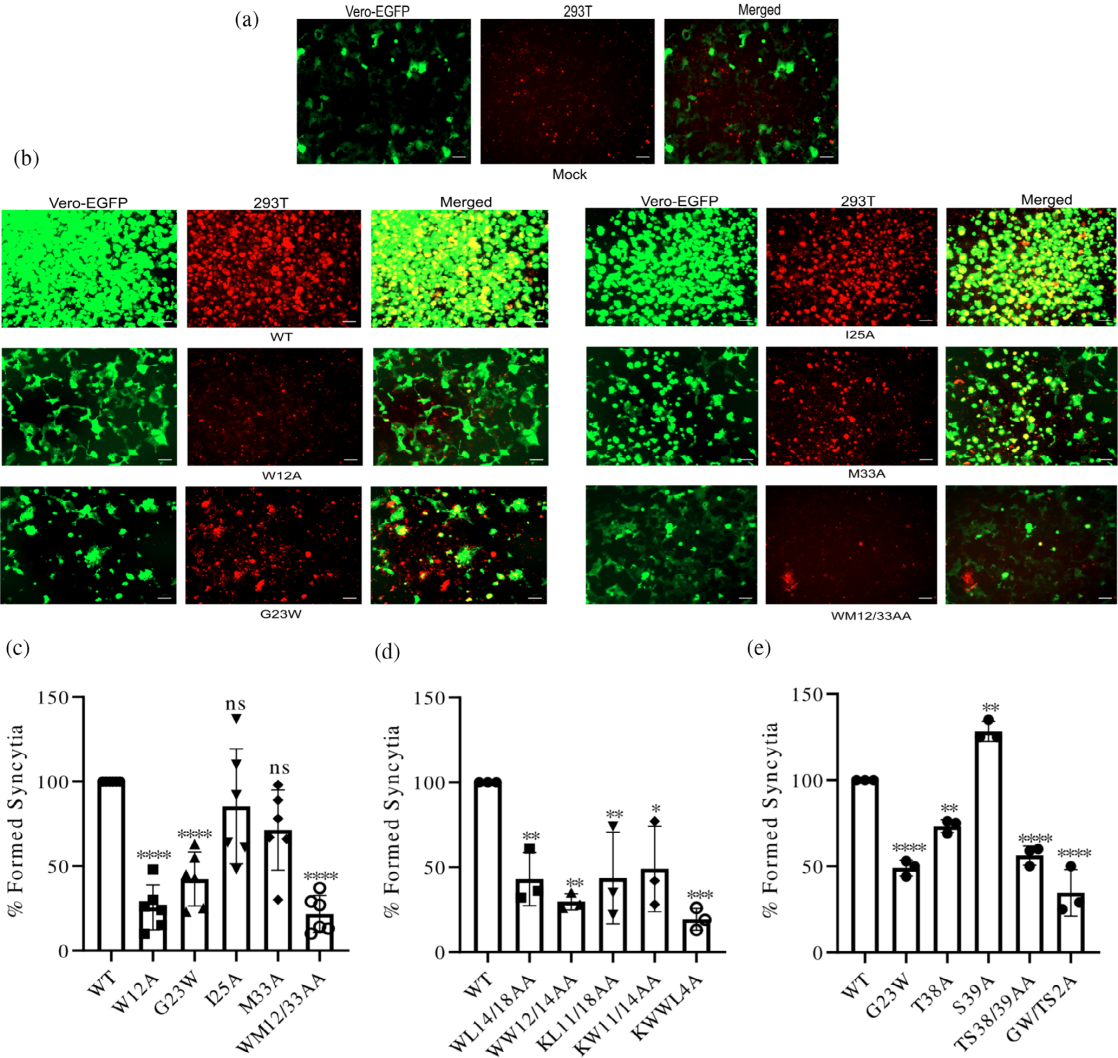
Impact of spike's transmembrane domain and cytoplasmic tail mutations on membrane fusion. (a, b) Syncytia visualization: wild‐type (WT) or mutant spike‐expressing 293T cells (b), along with untransfected control cells (Mock) (a), were stained with CellTracker Red CMTPX Dye (red) and overlaid onto Vero‐EGFP cells (green). Syncytia appear as yellow regions in merged images, indicating fusion between transfected and target cells (b). The scale bar is 2000 μm. Apparent differences in cell density reflect spike‐mediated fusion (W12A/G23W form few, small, dispersed syncytia while WT forms larger, clustered/partially detached syncytia) rather than unequal seeding. (c–e) Syncytia Quantification: The number of syncytia formed by each mutant was normalized to the number of syncytia that were hypothetically expected to form and expressed relative to the WT spike, which served as the reference and was defined as 100%. Results from six (c) or three (d and e) independent experiments were analyzed for mean ± SD. Statistical analysis was performed using one‐way analysis of variance followed by Dunnett's multiple comparison test. ns, not significant *p < 0.05, **p < 0.01, ***p < 0.001, ****p < 0.0001.

To assess how reduced palmitoylation affects the spike protein's ability to mediate membrane fusion, a syncytia formation assay was conducted. This assay utilized dual fluorescence imaging to visualize and quantify syncytia formation in transfected cells. Fused cells appeared as large, yellow structures due to the merging of red and green fluorescence. In the absence of spike expression, distinct red and green cells were observed, indicating the absence of cell fusion (Figure 10a).

Immunofluorescence images revealed a notable reduction in the fusogenic activity of several mutants, including G1223W, M1233A, and especially W1212A, as well as in the double mutant WM1212/33AA. In contrast, the mutant I1225A, which exhibited only a slight reduction in acylation, retained normal fusion capacity (Figure 10b).

Flow cytometry was used to quantify syncytia formation for each spike mutant, with fusion efficiency calculated as the proportion of dual‐positive (enhanced green fluorescent protein [EGFP]/red fluorescent protein) cells relative to the total number of transfected 293T cells (Figure S5). The resulting fusion efficiencies were normalized to the WT spike, set at 100%, which was included in each experiment as an internal control. The W1212A and G1223W mutations significantly reduced syncytia formation by 75% and 60%, respectively. In contrast, the M1233A mutation caused a modest, statistically insignificant reduction of 30%, and its combination with W1212A produced a similar effect as W1212A alone, with a combined reduction to 20%. The I1225A mutant had minimal impact, retaining 81% of the spike's fusion efficiency (Figure 10c).

Analysis of additional mutants demonstrated a consistent and substantial reduction in syncytia formation, ranging from 52% to 70% for the double mutants, regardless of their effect on acylation. This pattern was observed in mutants with strong (KW1211/14AA and KL1211/18AA), moderate (WW1212/14AA), and even insignificant (WL1214/18AA) reductions in acylation. The quadruple mutant KWWL4A showed an even greater decrease, suggesting a cumulative effect (Figure 10d).

Conversely, mutations in the CT produced varying effects. T1238A mutation caused a minor, statistically insignificant reduction in fusion, while S1239A slightly enhanced it. However, the combination of both mutations resulted in a significant 44% reduction. Adding G1223W to this combination further lowered fusion efficiency to 64% (Figure 10e).

These findings highlight the complex interplay between palmitoylation and fusion activity. Mutations in T1238 and S1239 reduced acylation but minimally affected syncytia formation, while WL1214/18AA impaired fusion without significantly lowering acylation. Conversely, G1223W decreased both acylation and fusion, whereas I1225A showed negligible effects on either.

## 3 DISCUSSION

This study explored the role of the TMD in the palmitoylation of the SARS‐CoV‐2 spike protein and its relationship to membrane fusion. Using AlphaFold2, we generated a structural model of the SARS‐CoV‐2 spike's TMD and CT, which closely aligns with MD simulations in terms of intrahelical contacts and overall trimeric organization (Aliper et al., 2022). Both computational models, however, diverge from the nuclear magnetic resonance (NMR) structure of a truncated and mutated 16‐residue TMD peptide, particularly at the helix–helix interface. In the NMR model, residues G1219, G1223, and A1226 are fully lipid‐facing, while I1221, I1225, L1229, and L1233 form a hydrophobic core along the threefold axis, creating an unusual tetrad repeat that stabilizes the trimer. Additionally, the aromatic‐rich region appears unstructured (Fu & Chou, 2021). Despite these differences, both the NMR and computational models support a stable trimeric α‐helical TMD. In contrast, coarse‐grained multimerization analyses reveal a spectrum of trimeric conformations: a symmetric trimer stabilized by a LXXIVXXT interface (F1220, I1221, L1224, I1227, and V1228), and an asymmetric trimer organized via a GXXXG dimerization motif (G1219–G1223), with an AXXXA motif (A1222 and A1226) representing intermediate states. The monomeric TM helix (Y1215–C1236) emerges as highly dynamic, exhibiting not only bobbing and tilting within the bilayer but also changes in helicity at N‐ and C‐termini (Lall et al., 2025). These observations support that the TMD is not static but instead possesses inherent flexibility, enabling lateral and rotational movement within the membrane and hence exposure of individual residues.

Our mutational analysis identified six residues—K1211, W1212, G1223, and M1233 within the TMD, and T1238 and S1239 in the CT—that are essential for efficient spike protein palmitoylation (Table 1). The reduced acylation observed for these mutants persisted under continuous inhibition of depalmitoylation, confirming that the decrease reflected impaired acylation rather than accelerated turnover. Co‐IP assays demonstrated a strong correlation between impaired palmitoylation and reduced enzyme‐substrate complex formation. Specifically, mutants exhibiting the most pronounced acylation defects (G1223W, WM1212/33AA, and TS1238/39AA) displayed the weakest interactions with hDHHC20. These findings suggest that diminished palmitoylation primarily stems from disrupted enzyme–substrate complex formation rather than impaired fatty acid transfer.

Based on these results, we propose that the signal for efficient spike protein acylation comprises three key components: (i) K1211, located at the interface between the ectodomain and TMD, likely exposes its side chain to interact with acidic residues in the short luminal domains of hDHHC20 (Figure S6), facilitating initial recognition. Substitution of K1211 only modestly reduces spike–DHHC20 association despite strongly impairing palmitoylation, suggesting that the K1211 interaction is weak and transient, but nevertheless contributes to positioning the TMD and CT relative to the DHHC20 catalytic site. (ii) A stable trimeric transmembrane helix, where W1212, G1223, and M1233 reside along the trimer interface. Although no specific sequence within the TMD appears critical, substitutions at these positions likely compromise the structural integrity of the helix, impairing acylation. In contrast, mutations of conserved and outward‐facing TMD residues (I1225, W1214, and L1218) had negligible effects on palmitoylation, underscoring the importance of trimer stability over sequence specificity. (iii) Two hydroxy‐amino acids, T1238 and S1239, positioned between the first and second clusters of acylated cysteines in the CT. Notably, the combined mutation of M1237 and T1238 was recently shown to exert the most pronounced impact on spike S‐acylation among all tested variants, as demonstrated by a novel in vitro assay employing purified DHHC20 and peptide fragments derived from the spike's CT (Mondal et al., 2025). These residues may interact with a hydrophilic region on hDHHC20's surface (Figure S6), enhancing substrate binding corroborating predictions by Panina et al. (2022). Future investigations of targeted alterations of DHHC20 will be important to directly test and refine the proposed interaction interface.

In the same context, we examined the role of DHHC9, another acyltransferase implicated in spike palmitoylation (Mesquita et al., 2021). None of the TMR mutants that reduced DHHC20 binding affected spike–DHHC9 association, which remained comparable to WT. This contrast highlights distinct recognition modes: DHHC20 interaction is sequence‐dependent, consistent with its role in initiating acylation at the two juxtamembrane cysteines that carry the majority of spike‐linked fatty acids, whereas DHHC9 binding is insensitive to TMR mutations and likely relies on structural context or localization. These findings refine the cooperative model proposed by Mesquita et al. (2021), in which DHHC20 catalyzes the initial modification at juxtamembrane sites to orient the CT, enabling DHHC9 to subsequently access and acylate distal cysteines.

Employing a similar approach, we previously investigated the signal governing the acylation of the influenza virus M2 ion channel, which carries a single palmitoylated cysteine within an amphiphilic helix near the TMD. In this system the DHHC20‐mediated acylation relies on the helix's biophysical properties rather than specific sequence motifs. Its tetrameric TMD contains a conserved glycine that introduces a kink, which slightly hinders the alignment of the target cysteine, positioning them proximal to the DHHC motif and modulating its catalytic efficiency (Meng et al., 2023).

The SARS‐CoV‐2 spike shares structural and functional features with influenza hemagglutinin (HA). Both influenza A and B HAs are acylated at CT cysteines, but by distinct DHHC enzymes: influenza A primarily by DHHC15 and DHHC20, and influenza B by ER‐localized DHHC2, DHHC4, and DHHC6 (Gadalla et al., 2020; Meng & Veit, 2023). Sequence features that promote acylation of the SAS‐CoV‐2 spike—methionine and hydroxy‐amino acids near acylation sites, and aromatic residues in the outer TMD—are present in both A and B HAs. Influenza A HAs, however, uniquely contain glycine residues within the TMD and a lysine between the TMD and ectodomain. Cryo‐EM analysis of full‐length H5 HA shows this lysine is surface‐exposed, suggesting a role in protein–protein interactions, while the unresolved C‐terminal region likely reflects splaying of polypeptide chains at the conserved glycine within the trimeric TMD (Benton et al., 2018). The conservation of glycine and lysine across all HA subtypes, together with their structural positioning, indicates that these residues may contribute to selective recognition by DHHC20 (Figure S7).

Multiple studies have investigated how mutations of palmitoylated cysteine residues in the SARS‐CoV‐2 spike protein influence viral entry and cell–cell fusion. The extent of functional loss depends on the number and position of mutated cysteines, with juxtamembrane residues exerting the strongest effect. Reported reductions range from approximately 4‐ to 40‐fold decreases in pseudovirus entry to 70%–100% loss of syncytia formation. Substitution of the entire cysteine cluster causes even greater impairment of infectivity and completely abolishes cell–cell fusion (Mesquita et al., 2021; Puthenveetil et al., 2021; Wu et al., 2021; Li, Liu, & Zhang, 2022; Ramadan et al., 2022).

In our assay, syncytia formation was reduced by up to 80%. The mutants retained high expression and plasma membrane trafficking consistent with partial acylation and explaining their milder fusion defects compared with full cysteine‐block mutants (Wu et al., 2021). Because virion and virus‐like particle assays depend on proper spike incorporation, they may reveal stronger effects of reduced palmitoylation.

Our fusion and acylation data reveal a nuanced relationship between spike palmitoylation and fusogenicity that can be separated into four patterns. (i) Some substitutions (e.g., I1225A) have essentially no effect on either acylation or fusion. (ii) A few mutations (e.g., G1223W) produce comparable reductions in both acylation and fusion, consistent with a direct dependence of the fusion activity on lipidation. (iii) Several mutants (e.g., M1233A, KL1211/18AA, KW1211/14AA, T1238A, S1239A, TS1238/39AA, and GW/TS2A) reduce total acylation more than they impair fusion. This implies that residual modification—either at a subset of critical cysteines or above a minimal “threshold” level of palmitoylation—can be sufficient to support fusion. The full‐length post‐fusion structure (Shi et al., 2023) places the juxtamembrane cysteine cluster roughly parallel to the membrane, with individual cysteines occupying distinct local environments, so palmitoylation of different residues could have non‐equivalent functional effects. (iv) Finally, a group of mutations (e.g., W1212A, WM1212/33AA, WW1212/14AA, WL1214/18AA, and KWWL4A) impair fusion much more than they reduce total acylation, implying additional palmitoylation‐independent effects. The Shi et al. structure supports this: W12 is positioned to contact the fusion peptide and may facilitate the conformational transitions required for membrane fusion (Figure S8), whereas W14 and L18 are membrane‐facing and could modulate local lipid, particularly cholesterol, interactions. These specific structural roles provide plausible mechanisms for loss of fusogenicity that are not simply explained by changes in overall palmitoylation. Combining structural mutations with cysteine substitutions would further help dissect the relationship between spike palmitoylation and fusion.

In conclusion, this study reveals the critical role of specific TMD and CT residues in mediating SARS‐CoV‐2 spike protein palmitoylation and its nuanced impact on membrane fusion. These findings advance our knowledge of the biophysical and structural interplay governing DHHC20‐mediated modifications and offer insights into the complex mechanisms underlying SARS‐CoV‐2 pathogenesis.

## 4 MATERIALS AND METHODS

### 4.1 Cell lines

Human embryonic kidney (HEK) 293T cells (American Type Culture Collection [ATCC] CRL‐11268) were used for transfection, S‐acylation, Co‐IP, and immunofluorescence studies. Vero E6 (ATCC CRL‐1586) and Vero E6‐EGFP cells (gift from Dr. Thomas Höfler, Kansas State University, Manhattan, Kansas, USA) were used, together with 293T cells, for syncytia formation assays. All cells were cultured in Dulbecco's Modified Eagle's Medium (DMEM, PAN Biotech) with 10% fetal bovine serum (FBS), 100 U/mL penicillin, and 100 μg/mL streptomycin at 37°C with 5% CO_2_.

### 4.2 Plasmids

A pUC57 plasmid with a human codon‐optimized SARS‐CoV‐2 spike gene (gift from Dr. Dusan Kunec, Max Delbrück Center, Berlin, Germany) was used. Spike mutations were introduced via quick‐change site‐directed mutagenesis (Wang & Malcolm, 2002), verified by Sanger sequencing, and cloned into pCAGGS for expression using enzyme‐based restriction cloning (Mikić et al., 2023). C‐terminal myc‐tagged human DHHC20 and HA‐tagged mouse DHHC9 in pcDNA3.1 were provided by the Fukata Lab (National Institutes for Physiological Sciences, Okazaki, Japan).

### 4.3 Antibodies

Primary antibodies included anti‐SARS‐CoV‐2 spike S2 subunit (GeneTex, GTX632604), anti‐flotillin‐2 (BD Biosciences, 610383), anti‐myc‐tag (Proteintech, 10828‐1‐AP), anti‐HA‐tag (Cell Signaling, 2367), and anti‐58K Golgi marker (Abcam, ab129005). Secondary antibodies were horseradish peroxidase (HRP)‐conjugated goat anti‐mouse (Bio‐Rad, 1706516) and anti‐rabbit (Bio‐Rad, 1706515) for Western blotting. For immunofluorescence, Alexa Fluor 488‐conjugated goat anti‐mouse (Invitrogen, #A28175) and goat anti‐rabbit (Invitrogen, #A11008), as well as Alexa Fluor 568‐conjugated goat anti‐mouse (Invitrogen, #A11004) and goat anti‐rabbit (Invitrogen, #A11011) antibodies were used.

### 4.4 Reagents

Transfections used Lipofectamine 3000 (ThermoFisher, L3000015) and Opti‐MEM (ThermoFisher, 31985070). Acyl‐RAC assays involved Thiopropyl Agarose (Creative Biomart, Thio‐001A), (Sigma, 208795), and Tris(2‐carboxyethyl)phosphine (TCEP, Carl Roth, HN95.2). Palmostatin B (Sigma‐Aldrich, 178501) was used for depalmitoylation inhibition, and protease inhibitor (Roche/Merck, 11873580001) was used in all protein experiments. Confocal microscopy employed ProLong Glass antifade mountant (ThermoFisher, P36984) and 4',6‐diamidino‐2‐phenylindole (ThermoFisher, 62247). Mutagenesis used S7 Fusion Polymerase (Mobidiag, MD‐S7). Co‐IP used NP‐40 lysis buffer (ThermoFisher, 85124) and Protein‐G Sepharose (GE Healthcare, GE17‐0618‐01). Western blots were developed with Pierce ECLplus (ThermoFisher, 32132). Flow cytometry used Zombie Violet™ (Biolegend, 423113), and syncytia assays used CellTracker Red CMTPX Dye (Invitrogen, C34552).

### 4.5 Acyl‐resin‐assisted capture

Spike protein acylation was measured as described (Abdulrahman & Veit, 2025). In brief, 293T cells (70%–90% confluent in six‐well plates) were transfected with 3 μg of WT or mutated spike plasmids per well. After 48 h, cells were washed with phosphate buffered saline (PBS) and lysed in 500 μL buffer A (1.5% Triton X‐100, 25 mM 4‐(2‐Hydroxyethyl)‐1‐piperazineethanesulfonic acid (HEPES) [pH 7.4], 25 mM NaCl, 1 mM ethylenediaminetetraacetic acid (EDTA), protease inhibitor cocktail). For the acyl‐RAC assay, non‐acylated cysteines were first reduced with 50 mM TCEP for 1 h at room temperature (RT), then blocked with methyl methanethiosulfonate (MMTS; 1.5% v/v in 100 mM HEPES, 1 mM EDTA, 87.5 mM SDS) at 40°C for 4 h. Proteins were precipitated overnight using 3–4 volumes of ice‐cold acetone at −20°C, pelleted (5000 × g, 10 min), washed four times with 70% acetone, air‐dried, and resuspended in 500 μL binding buffer (100 mM HEPES, 1 mM EDTA, 35 mM SDS, protease inhibitors).

Ten percent of each sample was reserved for total protein analysis by Western blot. The remainder was divided into two aliquots, treated either with 0.5M hydroxylamine (pH 7.4) to cleave thioester bonds or 0.5M Tris–HCl (pH 7.4) as a control and incubated with 50 μL pre‐washed Thiopropyl Agarose beads overnight at RT to capture free SH groups. Bound proteins were washed with binding buffer, eluted with 2× reducing sodium dodecyl sulfate‐polyacrylamide gel electrophoresis (SDS‐PAGE) buffer for 10 min at 95°C and centrifuged at 5000 rpm for 5 min. After SDS‐PAGE, Western blotting on difluoride membranes (GE Healthcare) was done using a voltage of 100 for 1 h. Membranes were blocked with 5% skim milk in PBST (PBS with 0.1% Tween‐20) for 1 h at RT, then incubated overnight at 4°C with primary antibodies diluted 1:1000. Primary antibodies targeted SARS‐CoV‐2 spike, flotillin‐2, and HA‐tag and myc‐tag. After three PBST washes, membranes were incubated for 1 h at RT with HRP‐conjugated secondary antibodies which are: goat anti‐mouse (1:3000) for spike and HA‐tag detection, goat anti‐mouse (1:5000) for flotillin‐2 and goat anti‐rabbit (1:3000) for the myc‐tag. All antibodies for Western blot were diluted in PBS containing 3% bovine serum albumin (BSA) and 0.1% Tween. Following three additional washes, membranes were developed using Pierce ECLplus reagent according to manufacturer's instructions and imaged with a Fusion SL camera system (Peqlab, Erlangen, Germany). Band densities were quantified using ImageJ v1.54f for acylation analysis (Schneider et al., 2012).

### 4.6 Inhibition of acyl‐protein thioesterase 1‐dependent depalmitoylation

To inhibit APT‐mediated depalmitoylation, HEK 293T cells (~70% confluent, six‐well plates) were transfected with 3 μg of WT spike expression plasmid per well and treated 4 h later with increasing concentrations of Palmostatin B (5–100 μM, dissolved in dimethylsulfoxid [DMSO]) or DMSO alone as a vehicle control to determine the optimal dose for maximal inhibition with minimal cytotoxicity. Palmostatin B was maintained throughout spike expression by re‐adding the respective concentrations 24 h after transfection. After 48 h, cells were harvested and analyzed by acyl‐RAC and Western blot as described above. Quantification of spike acylation relative to the DMSO control identified 37 μM Palmostatin B as the most effective concentration, providing maximal inhibition with acceptable cell viability; this concentration was used in all subsequent experiments.

For inhibition studies, cells were treated with 37 μM Palmostatin B or DMSO alone following transfection of WT or mutant spike constructs. Forty‐eight hours post‐transfection, lysates were processed for acyl‐RAC to assess spike palmitoylation as described above.

### 4.7 Co‐immunoprecipitation assay

To investigate interactions of SARS‐CoV‐2 spike proteins with DHHC20 and DHHC9, Co‐IP assays were conducted in 293T cells. Cells at 70% confluency in six‐well plates were co‐transfected with 3 μg of WT or mutant spike constructs and 2 μg of C‐terminal myc‐tagged DHHC20 or HA‐tagged DHHC9. After 48 h, cells were lysed in 300 μL of 1% NP‐40 buffer with protease inhibitors for 1 h on ice. Supernatants were collected, with 10% analyzed by Western blot using anti‐S2 and anti‐myc or anti‐HA antibodies to verify protein expression.

For immunoprecipitation, the remaining lysate was incubated with 1 μL anti‐myc or anti‐HA antibodies for 1 h at 4°C with gentle rotation, followed by overnight incubation with 50 μL protein‐G Sepharose beads. Beads were washed three times with 1% NP‐40 buffer, and captured proteins were eluted in 40 μL 2× SDS‐PAGE buffer. Eluates were analyzed by Western blot, and membranes were blotted with anti‐spike and anti‐myc or anti‐HA antibodies. The co‐precipitated spike protein levels were normalized to DHHC20 or DHHC9 bands and compared to WT spike to assess relative binding efficiency of mutants to the two acylating enzymes.

### 4.8 Syncytia formation assay

To assess syncytia formation, 293T cells at 50% confluency were transfected with 3 μg of plasmids encoding WT or mutant spike proteins. After 24 h, transfected or mock cells were co‐cultured at a 1:1 ratio with Vero E6‐EGFP cells for 48 h. Cells were detached with prewarmed 1× citric saline, washed twice with fluorescence‐activated cell sorting (FACS) buffer (2% FBS in PBS), and stained with anti‐SARS‐CoV‐2 spike antibody (1:500) overnight on ice. Another cell wash was followed by incubation with Alexa Fluor 568‐conjugated secondary antibody (1:1000) for 1 h on ice in the dark. After two additional washes, cells were resuspended in FACS buffer and analyzed using a CytoFLEX system (Beckman Coulter Life Sciences, USA). Flow cytometry was used to quantify spike‐mediated syncytia formation between Alexa Fluor 568‐labeled 293T donor cells (detected via secondary antibody fluorescence) and EGFP‐expressing Vero E6 target cells. Data were analyzed using a standardized gating strategy to ensure accurate identification of fused versus unfused cells. First, cell populations were gated on forward scatter (FSC)‐H versus side scatter (SSC)‐H to exclude debris and doublets were removed by gating singlets on FSC‐H versus FSC‐A. Next, spike‐positive donor cells were gated based on red fluorescence, while fused cells were defined as dual‐positive events, which also displayed increased FSC/SSC consistent with larger fused structures. Fusion efficiency was calculated by dividing the number of dual‐positive events by the total number of Alexa Fluor 568^+^ donor cells, assuming each transfected cell can participate in at least one fusion event. Each dual‐positive event was counted as one fusion event, regardless of size or number of participating cells. Fusion values for all mutants were normalized to WT spike analyzed in parallel. Vero E6 and non‐transfected 293T cells were used to define gating boundaries.

For immunofluorescence, 293T cells were stained 24 h post‐transfection with 0.5 μM CellTracker Red CMTPX Dye per manufacturer's instructions, then co‐cultured with Vero E6‐EGFP cells. Syncytia were visualized 48 h later using a ZEISS Colibri7 fluorescent microscope.

### 4.9 Spike surface abundance

To quantify spike protein surface abundance, 293T cells at ~70% confluency in six‐well plates were transfected with 3 μg of either WT or mutant spike expression plasmids. At 48 h post‐transfection, cells were washed with PBS, detached with 1× citric saline, washed twice, and stained with Zombie Violet™ (1:200) for 30 min in the dark to distinguish live from dead cells. For surface spike detection, cells were washed twice with FACS buffer, incubated with anti‐S2 antibody (1:500) overnight on ice, washed, and stained with Alexa Fluor 568‐conjugated secondary antibody (1:1000) for 1 h on ice.

For intracellular spike levels, cells were washed 3×, fixed with 4% paraformaldehyde (PFA) for 20 min, and permeabilized with 0.1% Triton X‐100 in PBS for 10 min. After washing, cells were incubated with anti‐S2 antibody (1:500) for 1 h, washed, and stained with Alexa Fluor 488‐conjugated secondary antibody (1:1000) for 30 min. Following a final wash, samples were analyzed by flow cytometry. The mean fluorescence intensity ratio of surface to intracellular spike protein was calculated for WT and mutant variants. The Zombie Violet™ stain and all antibodies were diluted in FACS buffer constituting 2% FBS in PBS.

### 4.10 AlphaFold2 structure prediction and analysis

The TMD and CT of the spike protein were predicted using the AlphaFold2advanced.ipynb notebook (https://colab.research.google.com/github/sokrypton/ColabFold/blob/v1.3.0/AlphaFold2.ipynb, accessed August 18, 2023). The following parameters were applied: “use_templates”: true, “num_relax”: 1, “msa_mode”: “mmseqs2_uniref_env,” “model_type”: “alphafold2_multimer_v3,” “num_models”: 5, “num_recycles”: null, “pair_mode”: “unpaired_paired,” “pairing_strategy”: “greedy,” “random_seed”: 0, “num_seeds”: 1. The amino acid sequence GKYEQYIKWPWYIWLGFIAGLIAIVMVTIMLCCMTSCCSCLKGCCSCGSCCKFDEDDSEPVLKGVKLHYT was submitted for structure prediction.

The resulting Protein Data Bank (PDB) file was oriented in a virtual lipid bilayer using the PPM 3.0 Web Server (https://opm.phar.umich.edu/ppm_server3_cgopm), accessed on August 1, 2024, configured with a flat plasma membrane and a hydrophobic thickness of 33 Å. Amino acid conservation within the TMD was analyzed and visualized using WebLogo (https://weblogo.berkeley.edu/logo.cgi) accessed on November 12, 2025. Forty‐one spike protein sequences with the following Uniport numbers were used: P15423, Q6Q1S2, Q0Q466, P24413, P27655, P07946, P18450, P33470, P10033, Q65984, Q7T6T3, P36300, A3EXG6, P0DTC2, P59594, Q3LZX1, Q3I5J5, Q0Q475, K9N5Q8, A3EXD0, Q0Q4F2, A3EX94, Q9IKD1, P11224, Q02385, P11225, P22432, P36334, Q9QAR5, Q9QAQ8, Q91A26, Q8V436, P15777, P25193, P25191, P25194, P25190, P25192, Q5MQD0, Q0ZME7, and Q14EB0 Structural visualization and analysis of the PDB files were performed using PyMOL version 2.1.1 (Molecular Graphics System; Schrödinger, LLC, NY, USA).

## AUTHOR CONTRIBUTIONS

**Dina A. Abdulrahman:** Conceptualization; investigation; writing – original draft; writing – review and editing. **Michael Veit:** Conceptualization; funding acquisition; writing – original draft; writing – review and editing; supervision.

## FUNDING INFORMATION

This work was supported by DFG project VE 141/20‐1 (awarded to Michael Veit). Dina A. Abdulrahman is a recipient of a Ph.D. scholarship from the Central Department of Missions (Egyptian Ministry of Higher Education).

## Supporting information


**Figure S1.** Confidence metrices for the predicted structure of the C‐terminus of the SARS‐CoV‐2 spike (A) Amino acid sequence used for the prediction. (B) Predicted local distance difference test (pLDDT) score per position for the five models generated by alphafold2. The amino acid position is plotted against the pLDDT. Values between 70 and 90 indicate a high accuracy, where the prediction of the main chain of the protein is reliable. Values between 50 and 70 indicate a lower accuracy, but it is likely that the predictions of individual secondary structures are correct. Values below 50 indicate that this part might be unstructured (C) The prediction aligned error (PAE) measures confidence in the relative positions of pairs of amino acids. PAE is displayed as a 2D plot and the expected position error in Angstrom is color‐coded. Both axes indicate the position of the individual amino acids. The uncertainty in the predicted distance of two amino acids is color‐coded from dark blue (0 Å) to deep red (30 Å), as shown in the side bar. The color of the intersection of a horizontal line drawn from the position of an amino acid on the y‐axis and a vertical line from the position of another amino acid on the x‐axis indicates the error in the predicted distance between these two residues. PAE graphs are always characterized by a diagonal blue line, since amino acids that are juxtaposed in the primary sequence are also adjacent in the 3D structure.

**Figure S2.** Surface expression of the spike mutants. (A) Flow cytometry gating strategy for spike surface and intracellular expression. Mock‐transfected cells (left) were used to define background fluorescence and establish gating thresholds. WT spike‐transfected cells (right) show distinct populations corresponding to intracellular (FITC^+^, green), surface (PE^+^, red), and double‐positive (FITC^+^/PE^+^, yellow) cells. Percentages indicate the proportion of each population among viable cells. The same gating strategy was applied to spike mutants for quantitative comparison. (B) The proportion of surface‐expressed spike protein for WT and various mutants is shown. The bars represent the WT and mutated spikes, with single and combined mutations indicated on the x‐axis. All mutants displayed surface expression levels comparable to the WT spike, indicating no significant defects in trafficking or membrane transport. Data represent mean ± SD from three independent experiments. Statistical analysis was performed using one‐way ANOVA followed by Dunnett's multiple comparison test.

**Figure S3.** Dose optimization of Palmostatin B for spike depalmitoylation inhibition. (A) Total extract (TE) of HEK 293T cells expressing WT spike protein treated with increasing concentrations of Palmostatin B (0–100 μM). S2 and S2′ are the spike cleavage products while Flotillin‐2 (FLOT2) served as a loading control. (B) Acyl‐RAC assay of the same samples showing dose‐dependent inhibition of APT‐mediated spike depalmitoylation. The banding pattern is as described in (A). Samples were treated with either hydroxylamine (+HA) to cleave cysteine‐bound fatty acids or Tris–HCl (−HA) to assess the specificity of the acylation test. (C) Quantification of spike S‐acylation of Palmostatin B‐treated samples by normalizing their band to their respective input bands and comparing them to untreated cells (set to 1). Palmostatin B at 37 μM demonstrates maximal inhibition without significant cytotoxicity. Data represent mean ± SD from three independent experiments. Statistical analysis was performed using one‐way ANOVA followed by Dunnett's multiple comparison test (ns, not significant; **p < 0.01; ****p < 0.0001).

**Figure S4.** Co‐IP analysis of spike–hDHHC20 and spike‐mDHHC9 interactions. (A, C) Western blot analysis of total protein extracts (Input) from HEK 293 T cells expressing only the spike, only hDHHC20‐myc (A) or hDHHC9‐HA (C), or co‐ expressing both constructs. Membranes were probed with anti‐S2 to detect full‐length (S0) and cleaved spike (S2, S2′) and with anti‐myc or anti‐HA to confirm expression of DHHC20 and DHHC9, respectively. (B, D) Co‐immunoprecipitation (Co‐IP) of spike–DHHC complexes. Lysates were immunoprecipitated using anti‐myc (B) or anti‐HA (D) antibodies, and co‐precipitated spike proteins were detected by immunoblotting with anti‐S2, while DHHC20‐myc or DHHC9‐HA were detected with anti‐myc or anti‐HA antibodies.

**Figure S5.** Flow cytometry gating strategy for syncytia formation assay: Representative dot plots showing the sequential gating approach applied to all experiments. Vero E6 (top left) and non‐transfected 293T (top middle) cells establish fluorescence thresholds and set the primary FITC and PE gates. The “Mock” control (top right) defines the quadrant boundaries used to distinguish Vero‐EGFP cells (FITC^+^/PE^−^), 293T‐568 cells (FITC^−^/PE^+^), double‐negative events (FITC^−^/PE^−^), and double‐positive syncytia (FITC^+^/PE^+^). The bottom row shows the corresponding single‐color controls for FITC‐only Vero‐EGFP cells (left) and PE‐only transfected 293T‐568 cells (middle). The WT condition (bottom right) illustrates the application of these gates to quantify Vero‐EGFP cells, 293T‐568 cells, double‐positive syncytia, and double‐negative populations.

**Figure S6.** Surface Representation of DHHC20. A semi‐transparent surface projection of DHHC20, generated from PDB file 6BML. Hydrophobic residues (ALA, VAL, LEU, ILE, PHE, TRP, PRO, TYR, GLY, MET) are colored orange, while hydrophilic residues (SER, THR, ASN, GLN, ASP, GLU, LYS, ARG, HIS, CYS) are shown in blue. A hydrophilic patch in the transmembrane region is formed by residues S223, S226, and S229. Acidic residues in the luminal domain (E46, E47, E191, E200) are highlighted as red spheres. The fatty acid attached to the Cys of the DHHC motif is shown as green spheres.

**Figure S7.** C‐terminal amino acid sequence and membrane‐anchor structure of influenza virus hemagglutinin (HA). (A) Consensus sequences of the 16 HA subtypes of Influenza A and of Influenza B, adapted from Siche et al. (2015). Amino acids relevant to SARS‐CoV‐2 spike acylation are highlighted in different colors: pink indicates basic residues in the outer region of the TMD, slate blue marks aromatic residues, gray denotes glycine located centrally within the TMD, light green highlights hydroxy‐amino acids, light blue represents methionine residues in the cytoplasmic tail, and yellow identifies acylated cysteine. (B) Cryo‐EM structure of the membrane‐anchor region of an H5 subtype HA (PDB: 6HJQ). Amino acids important for acylation of the spike of SARS‐CoV‐2 are shown as spheres. At the C‐terminal glycine, the polypeptide chains diverge, resulting in structural disorder and a lack of resolution in this region.

**Figure S8.** Post‐fusion structure of the spike of SARS‐CoV‐2. The membrane‐embedded region of the SARS‐CoV‐2 spike trimer in its post‐fusion conformation, derived from PDB file 8FDW. The transmembrane domain (TMD) and cytoplasmic tail (CT) are shown as green cartoons, the fusion peptide (FP) as cyan cartoons, with one FP from the same protomer highlighted in light blue. Amino acids involved in interactions (left) and those substituted in this study (right) are represented as sticks. Acylated cysteines in the CT are depicted as orange sticks.

## Data Availability

All data are included in the manuscript.
