# Supplementary figures and images for: Role of the transmembrane domain in severe acute respiratory syndrome (SARS) coronavirus 2 spike for palmitoylation and membrane fusion
Source: Protein Sci. 2026 Jan 23;35(2):e70482. doi: 10.1002/pro.70482 (PMC12828983; doi:10.1002/pro.70482)

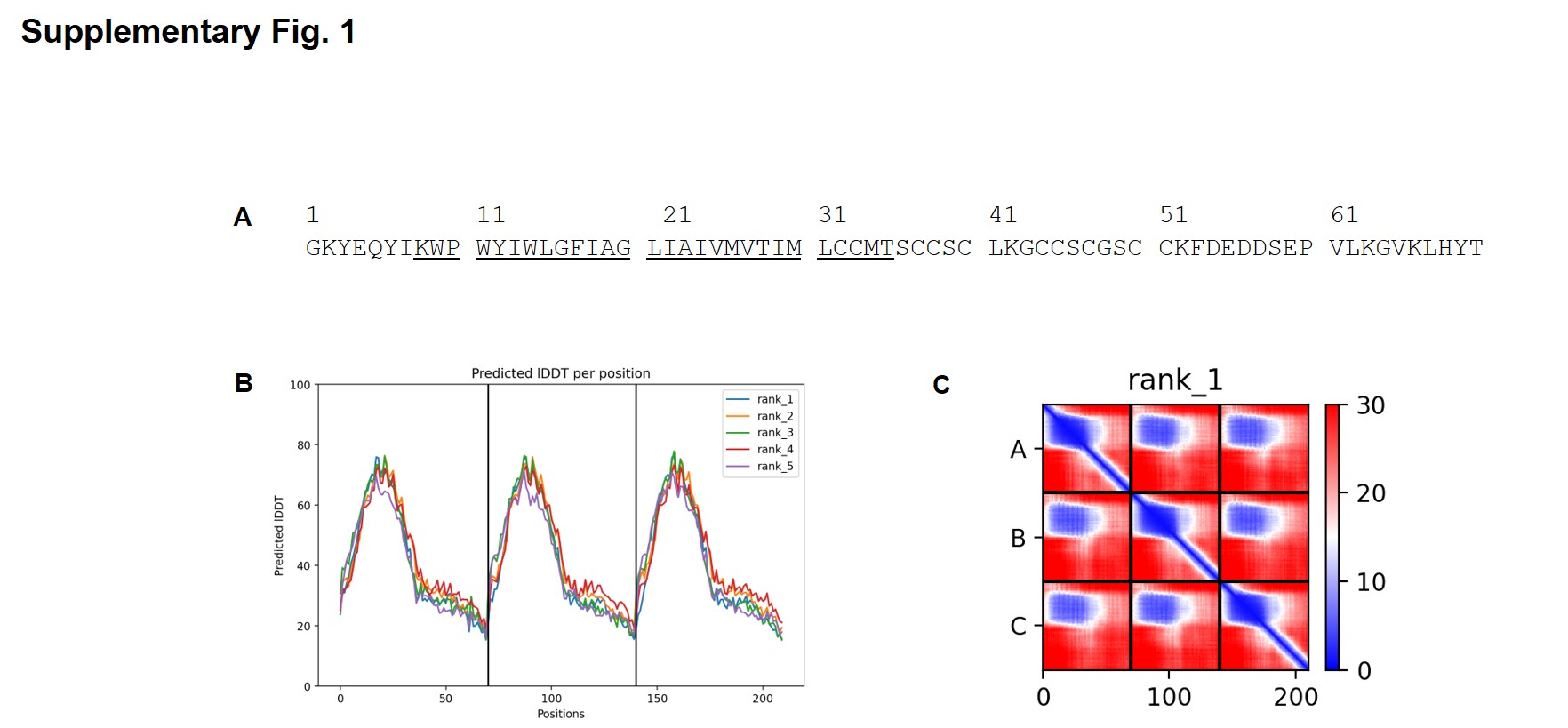

Supplement: Supplementary file 1 — Figure S1. Confidence metrices for the predicted structure of the C‐terminus of the SARS‐CoV‐2 spike (A) Amino acid sequence used for the prediction. (B) Predicted local distance difference test (pLDDT) score per position for the five models generated by alphafold2. The amino acid position is plotted against the pLDDT. Values between 70 and 90 indicate a high accuracy, where the prediction of the main chain of the protein is reliable. Values between 50 and 70 indicate a lower accuracy, but it is likely that the predictions of individual secondary structures are correct. Values below 50 indicate that this part might be unstructured (C) The prediction aligned error (PAE) measures confidence in the relative positions of pairs of amino acids. PAE is displayed as a 2D plot and the expected position error in Angstrom is color‐coded. Both axes indicate the position of the individual amino acids. The uncertainty in the predicted distance of two amino acids is color‐coded from dark blue (0 Å) to deep red (30 Å), as shown in the side bar. The color of the intersection of a horizontal line drawn from the position of an amino acid on the y‐axis and a vertical line from the position of another amino acid on the x‐axis indicates the error in the predicted distance between these two residues. PAE graphs are always characterized by a diagonal blue line, since amino acids that are juxtaposed in the primary sequence are also adjacent in the 3D structure. [file PRO-35-e70482-s004.jpg]

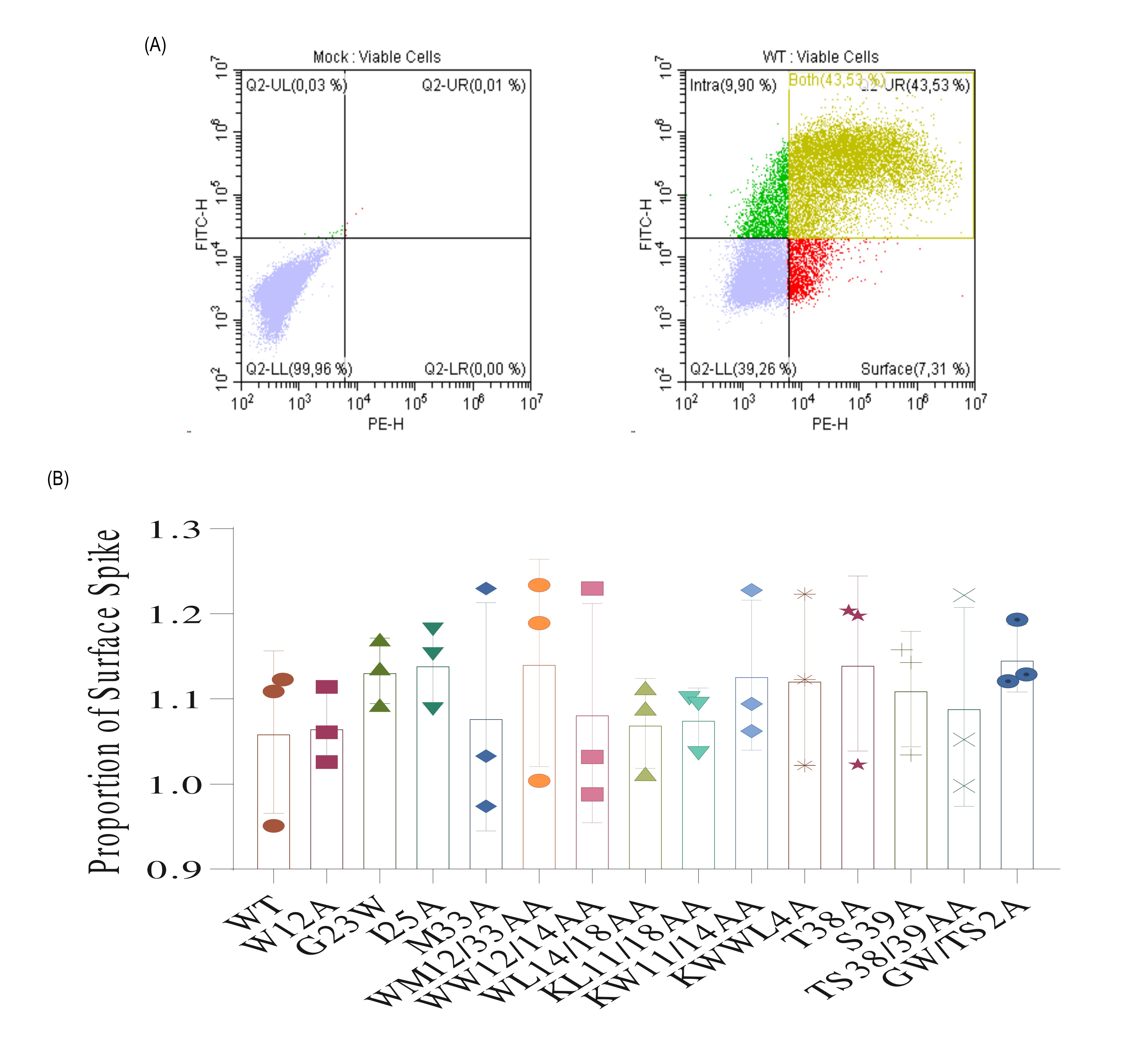

Supplement: Supplementary file 2 — Figure S2. Surface expression of the spike mutants. (A) Flow cytometry gating strategy for spike surface and intracellular expression. Mock‐transfected cells (left) were used to define background fluorescence and establish gating thresholds. WT spike‐transfected cells (right) show distinct populations corresponding to intracellular (FITC+, green), surface (PE+, red), and double‐positive (FITC+/PE+, yellow) cells. Percentages indicate the proportion of each population among viable cells. The same gating strategy was applied to spike mutants for quantitative comparison. (B) The proportion of surface‐expressed spike protein for WT and various mutants is shown. The bars represent the WT and mutated spikes, with single and combined mutations indicated on the x‐axis. All mutants displayed surface expression levels comparable to the WT spike, indicating no significant defects in trafficking or membrane transport. Data represent mean ± SD from three independent experiments. Statistical analysis was performed using one‐way ANOVA followed by Dunnett's multiple comparison test. [file PRO-35-e70482-s008.jpg]

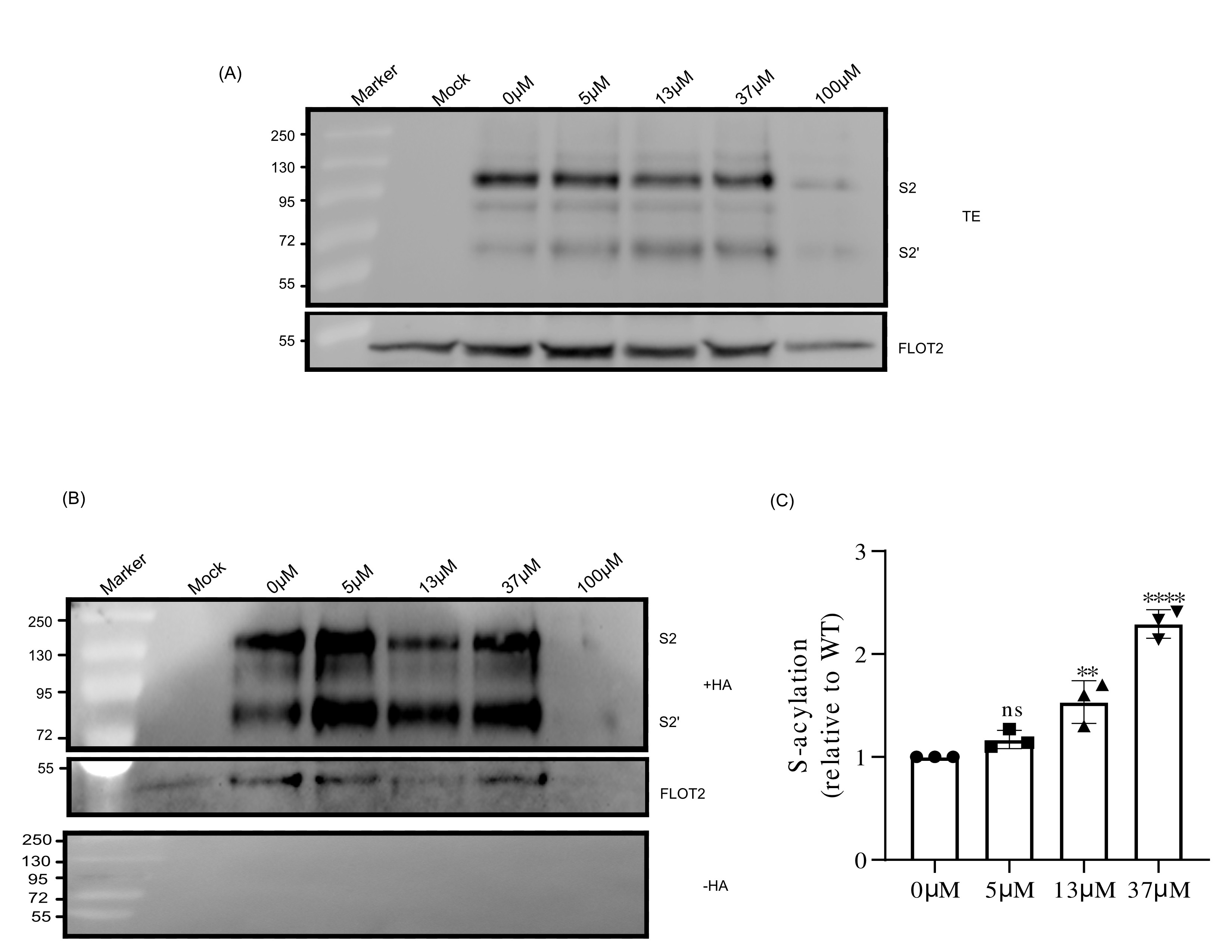

Supplement: Supplementary file 3 — Figure S3. Dose optimization of Palmostatin B for spike depalmitoylation inhibition. (A) Total extract (TE) of HEK 293T cells expressing WT spike protein treated with increasing concentrations of Palmostatin B (0–100 μM). S2 and S2′ are the spike cleavage products while Flotillin‐2 (FLOT2) served as a loading control. (B) Acyl‐RAC assay of the same samples showing dose‐dependent inhibition of APT‐mediated spike depalmitoylation. The banding pattern is as described in (A). Samples were treated with either hydroxylamine (+HA) to cleave cysteine‐bound fatty acids or Tris–HCl (−HA) to assess the specificity of the acylation test. (C) Quantification of spike S‐acylation of Palmostatin B‐treated samples by normalizing their band to their respective input bands and comparing them to untreated cells (set to 1). Palmostatin B at 37 μM demonstrates maximal inhibition without significant cytotoxicity. Data represent mean ± SD from three independent experiments. Statistical analysis was performed using one‐way ANOVA followed by Dunnett's multiple comparison test (ns, not significant; **p < 0.01; ****p < 0.0001). [file PRO-35-e70482-s001.jpg]

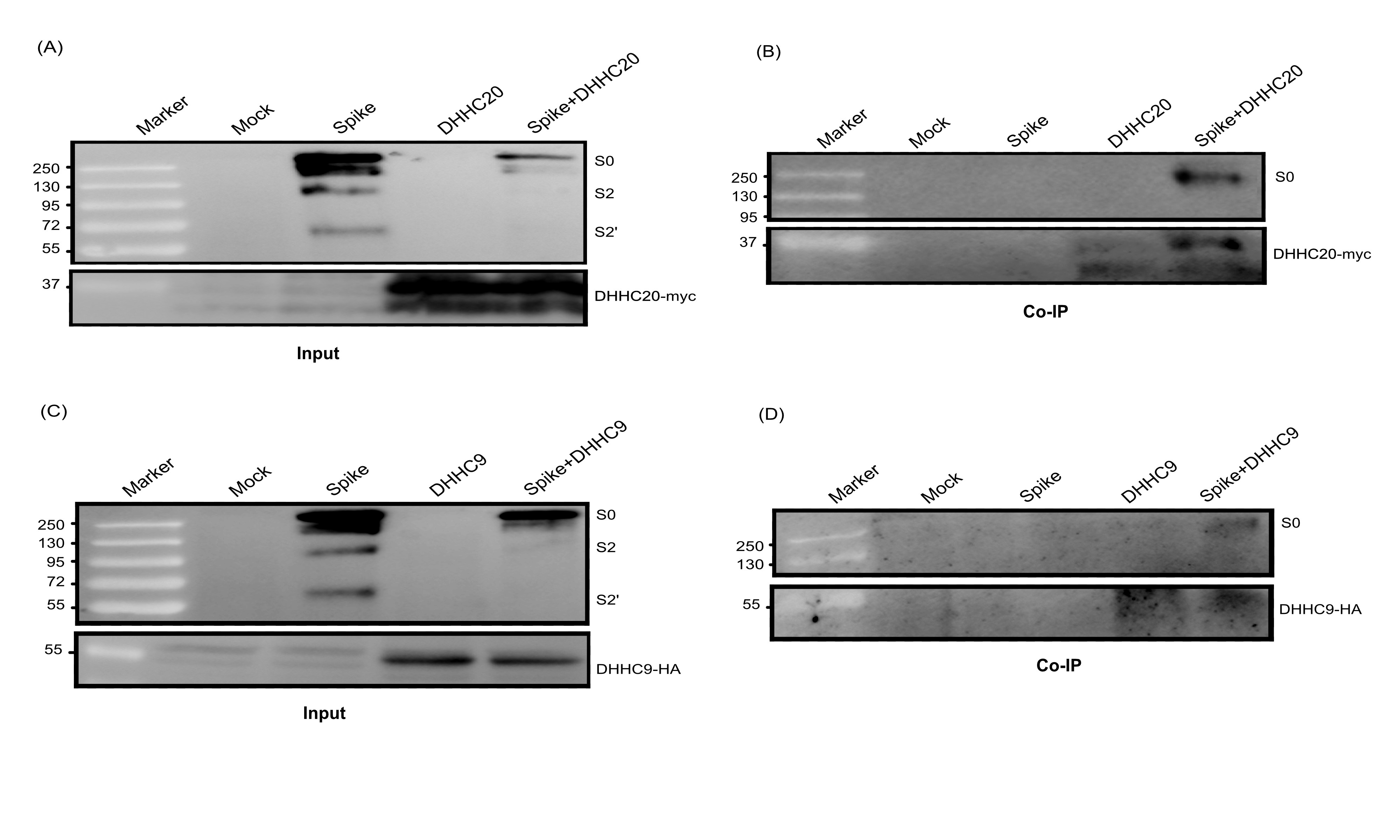

Supplement: Supplementary file 4 — Figure S4. Co‐IP analysis of spike–hDHHC20 and spike‐mDHHC9 interactions. (A, C) Western blot analysis of total protein extracts (Input) from HEK 293 T cells expressing only the spike, only hDHHC20‐myc (A) or hDHHC9‐HA (C), or co‐ expressing both constructs. Membranes were probed with anti‐S2 to detect full‐length (S0) and cleaved spike (S2, S2′) and with anti‐myc or anti‐HA to confirm expression of DHHC20 and DHHC9, respectively. (B, D) Co‐immunoprecipitation (Co‐IP) of spike–DHHC complexes. Lysates were immunoprecipitated using anti‐myc (B) or anti‐HA (D) antibodies, and co‐precipitated spike proteins were detected by immunoblotting with anti‐S2, while DHHC20‐myc or DHHC9‐HA were detected with anti‐myc or anti‐HA antibodies. [file PRO-35-e70482-s003.jpg]

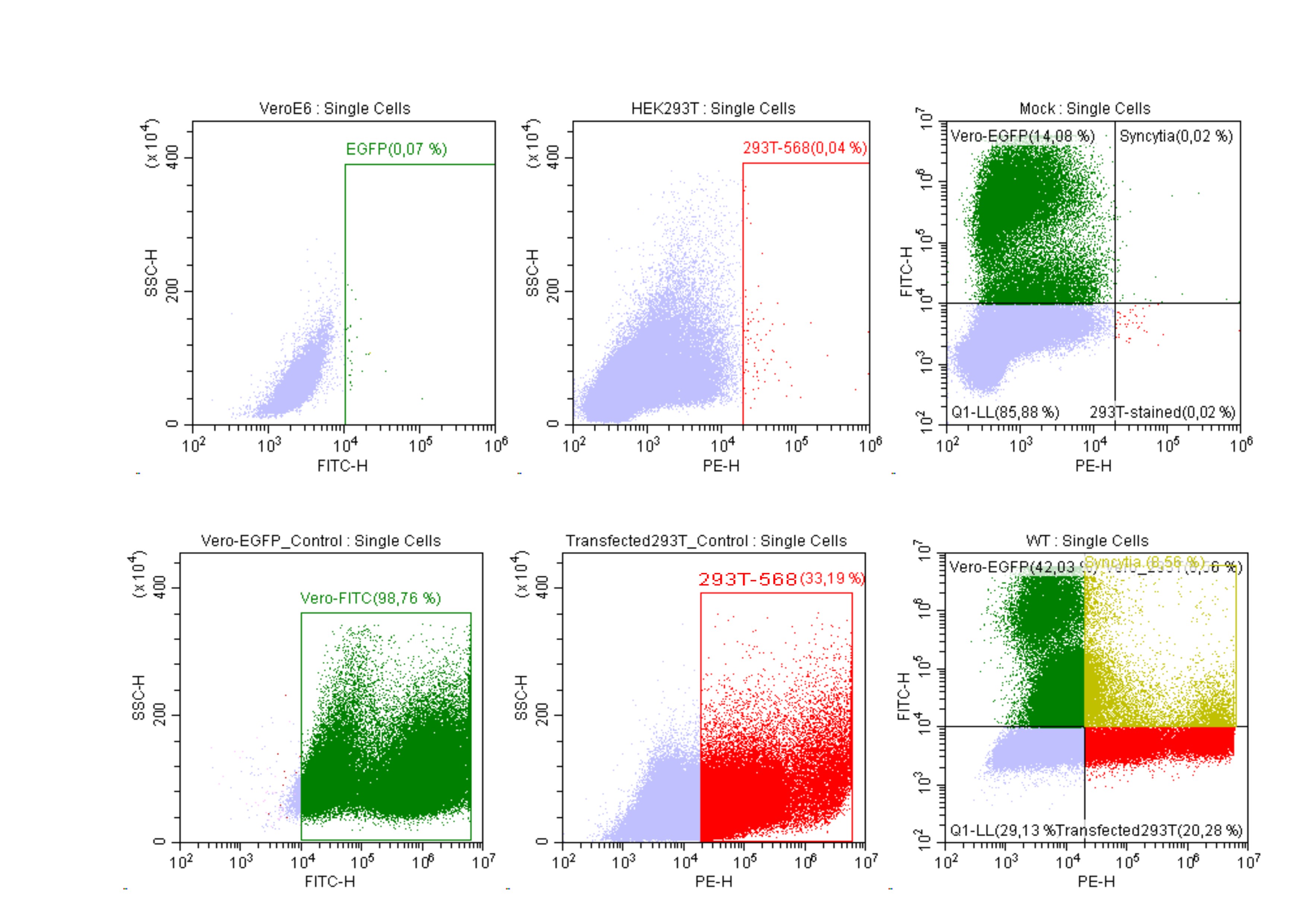

Supplement: Supplementary file 5 — Figure S5. Flow cytometry gating strategy for syncytia formation assay: Representative dot plots showing the sequential gating approach applied to all experiments. Vero E6 (top left) and non‐transfected 293T (top middle) cells establish fluorescence thresholds and set the primary FITC and PE gates. The “Mock” control (top right) defines the quadrant boundaries used to distinguish Vero‐EGFP cells (FITC+/PE−), 293T‐568 cells (FITC−/PE+), double‐negative events (FITC−/PE−), and double‐positive syncytia (FITC+/PE+). The bottom row shows the corresponding single‐color controls for FITC‐only Vero‐EGFP cells (left) and PE‐only transfected 293T‐568 cells (middle). The WT condition (bottom right) illustrates the application of these gates to quantify Vero‐EGFP cells, 293T‐568 cells, double‐positive syncytia, and double‐negative populations. [file PRO-35-e70482-s005.jpg]

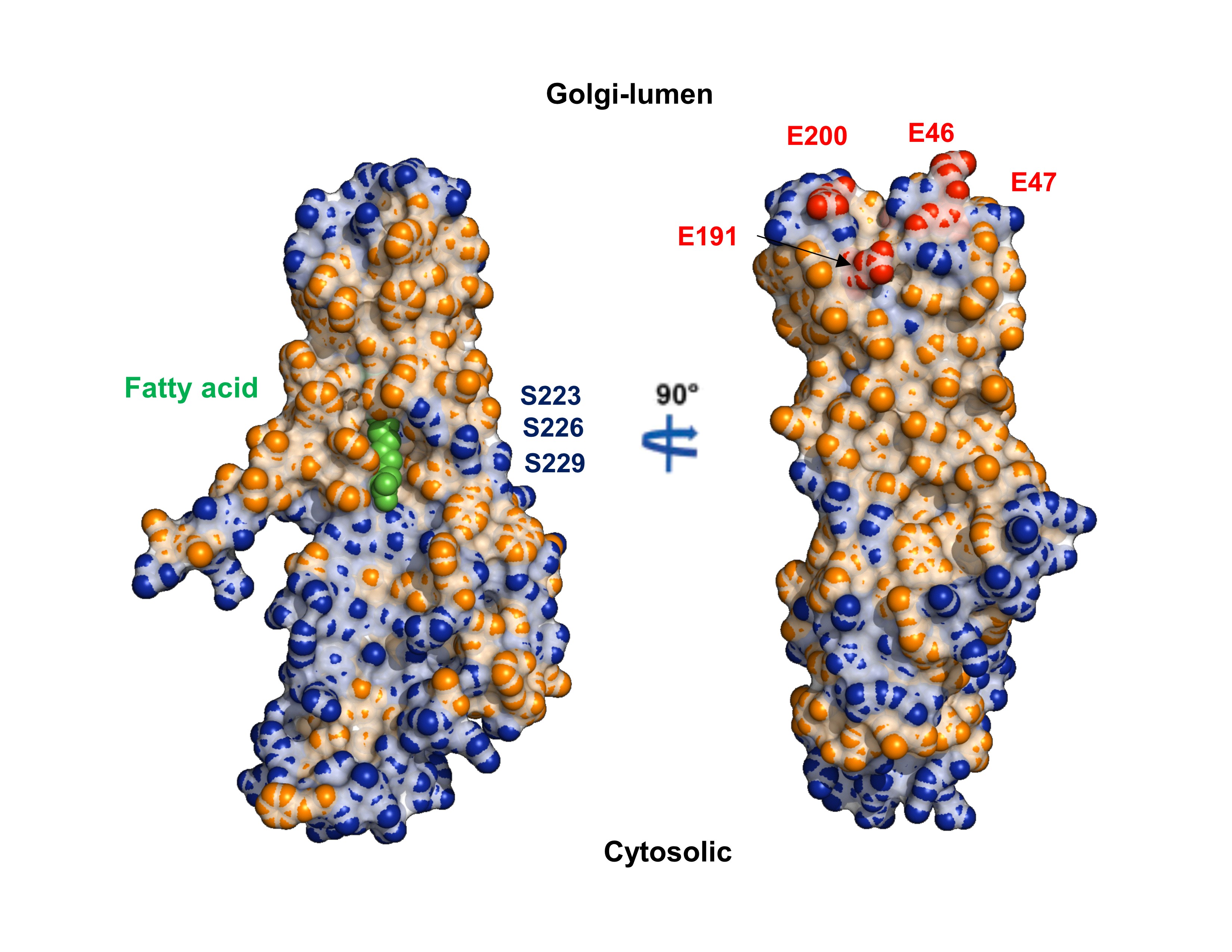

Supplement: Supplementary file 6 — Figure S6. Surface Representation of DHHC20. A semi‐transparent surface projection of DHHC20, generated from PDB file 6BML. Hydrophobic residues (ALA, VAL, LEU, ILE, PHE, TRP, PRO, TYR, GLY, MET) are colored orange, while hydrophilic residues (SER, THR, ASN, GLN, ASP, GLU, LYS, ARG, HIS, CYS) are shown in blue. A hydrophilic patch in the transmembrane region is formed by residues S223, S226, and S229. Acidic residues in the luminal domain (E46, E47, E191, E200) are highlighted as red spheres. The fatty acid attached to the Cys of the DHHC motif is shown as green spheres. [file PRO-35-e70482-s006.jpg]

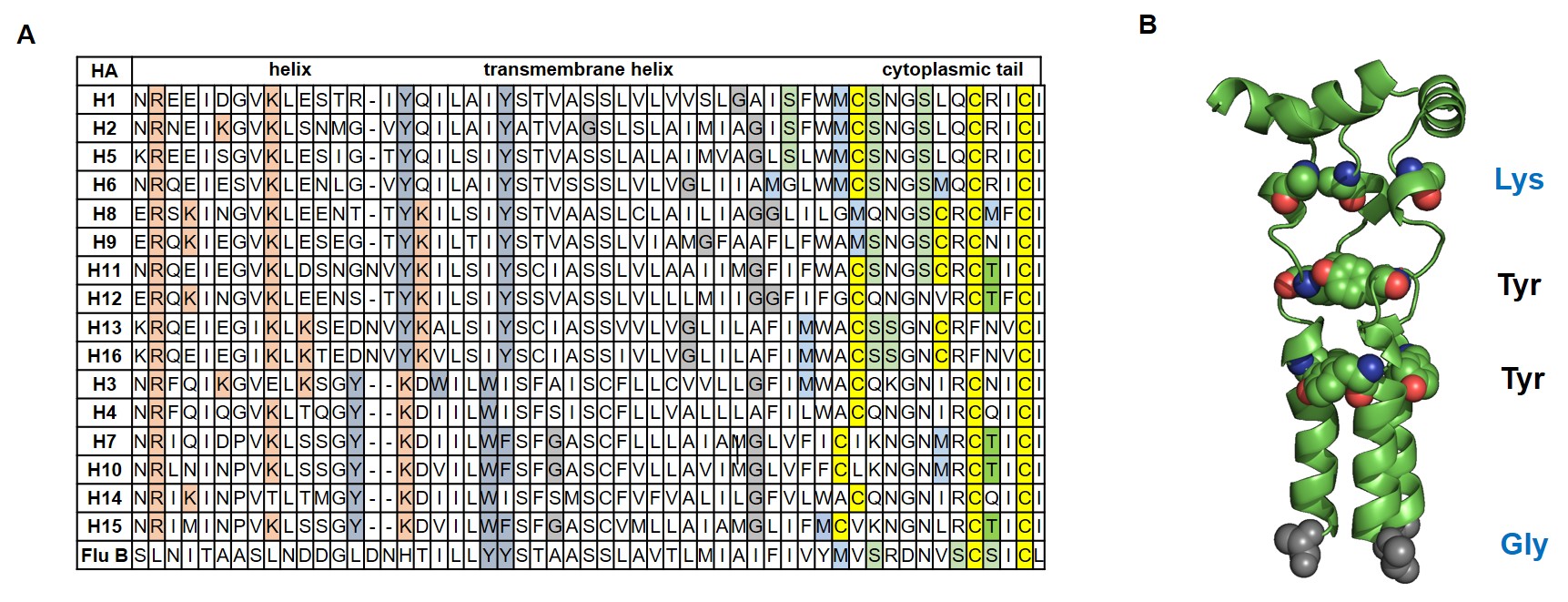

Supplement: Supplementary file 7 — Figure S7. C‐terminal amino acid sequence and membrane‐anchor structure of influenza virus hemagglutinin (HA). (A) Consensus sequences of the 16 HA subtypes of Influenza A and of Influenza B, adapted from Siche et al. (2015). Amino acids relevant to SARS‐CoV‐2 spike acylation are highlighted in different colors: pink indicates basic residues in the outer region of the TMD, slate blue marks aromatic residues, gray denotes glycine located centrally within the TMD, light green highlights hydroxy‐amino acids, light blue represents methionine residues in the cytoplasmic tail, and yellow identifies acylated cysteine. (B) Cryo‐EM structure of the membrane‐anchor region of an H5 subtype HA (PDB: 6HJQ). Amino acids important for acylation of the spike of SARS‐CoV‐2 are shown as spheres. At the C‐terminal glycine, the polypeptide chains diverge, resulting in structural disorder and a lack of resolution in this region. [file PRO-35-e70482-s002.jpg]

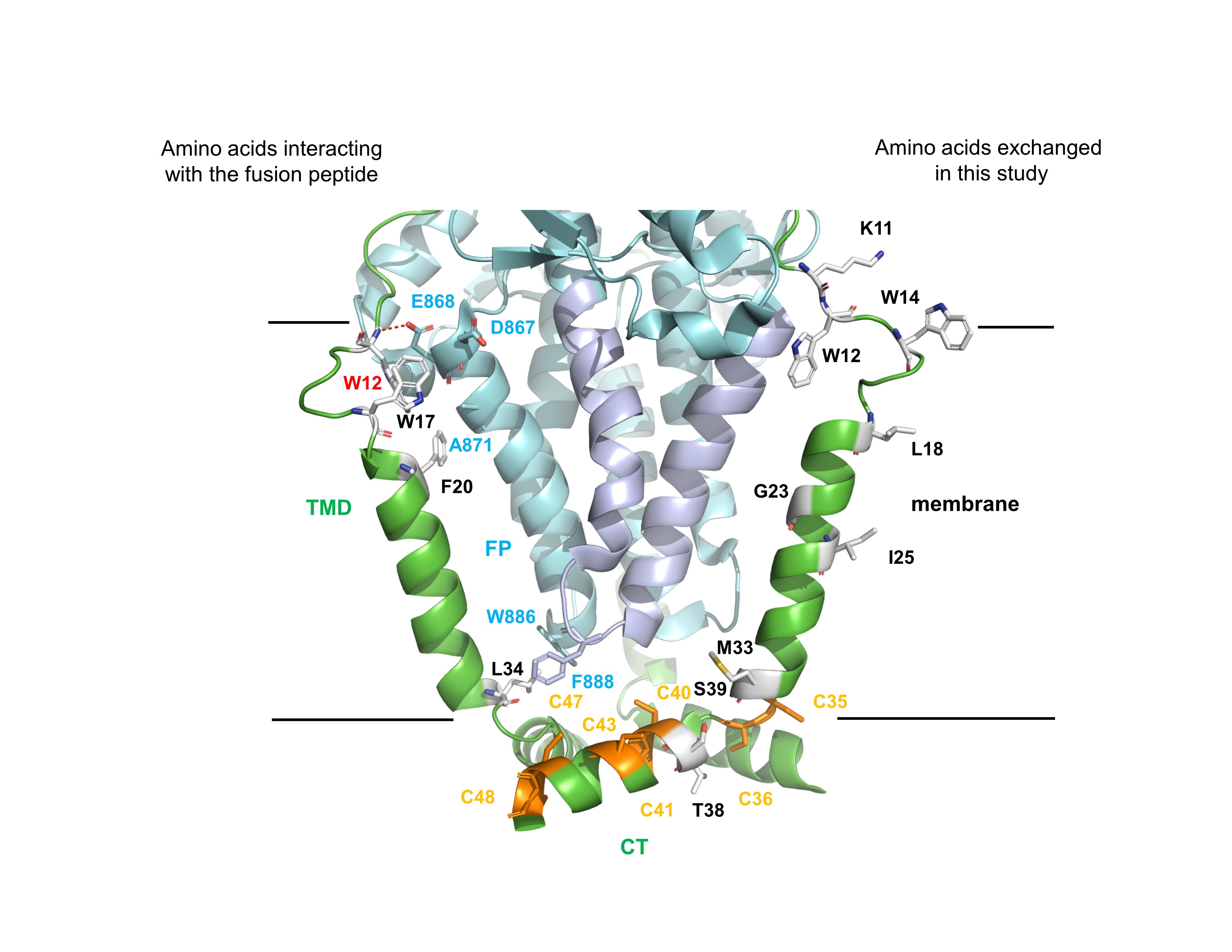

Supplement: Supplementary file 8 — Figure S8. Post‐fusion structure of the spike of SARS‐CoV‐2. The membrane‐embedded region of the SARS‐CoV‐2 spike trimer in its post‐fusion conformation, derived from PDB file 8FDW. The transmembrane domain (TMD) and cytoplasmic tail (CT) are shown as green cartoons, the fusion peptide (FP) as cyan cartoons, with one FP from the same protomer highlighted in light blue. Amino acids involved in interactions (left) and those substituted in this study (right) are represented as sticks. Acylated cysteines in the CT are depicted as orange sticks. [file PRO-35-e70482-s007.jpg]
